# *N,N*-Dimethylaminopyrene as a fluorescent affinity mass tag for ligand-binding mode analysis

**DOI:** 10.1038/s41598-020-64321-9

**Published:** 2020-04-30

**Authors:** Atsushi Arai, Rei Watanabe, Atsunori Hattori, Keita Iio, Yaping Hu, Kozo Yoneda, Hideo Kigoshi, Masaki Kita

**Affiliations:** 1 Graduate School of Bioagricultural Sciences, Nagoya University, Furo-cho, Chikusa, Nagoya 464-8601 Japan; 2 Graduate School of Pure and Applied Sciences, University of Tsukuba, 1-1-1 Tennodai, Tsukuba, 305-8571 Japan

**Keywords:** Mass spectrometry, Proteomic analysis, Chemical tools, Peptides, Proteins

## Abstract

Elucidation of the binding mode of protein–ligand interactions provides insights for the design of new pharmacological tools and drug leads. Specific labeling of target proteins with chemical probes, in which the ligands are conjugated with reacting and detecting groups, can establish the binding positions of ligands. Label-assisted laser desorption/ionization mass spectrometry (LA-LDI MS) is a promising detection method to selectively detect labeled molecules. However, previous LDI MS tags, such as nitrogen-substituted pyrenes, had problems with low sensitivity and stability. Here we show 6-*N*,*N*-dimethylaminopyrene (dmpy) as a versatile mass tag, which was detected at an amount of 0.1 fmol by LA-LDI MS and applicable for MS/MS analysis. By using ligand-dissociation-type dmpy probes and affinity purification with a polystyrene gel, we demonstrated that dmpy-labeled peptides were predominantly detected by MALDI MS. Our dmpy-probe-labeling method might be highly useful for determining the target biomacromolecules of various ligands and their binding sites.

## Introduction

To identify the target molecules of bioactive small molecules (ligands) and analyze their interactions are essential in the fields of medicinal chemistry and chemical biology^1–3^. An effective approach to such binding-mode analysis is to utilize chemical probes, in which the ligands are conjugated with reacting groups and detecting groups with the activity maintained^4,5^. In the case of proteins, enzymatic digestion (such as trypsin) and subsequent peptide-mass fingerprint (PMF) analysis or related peptide sequence analyses can determine the ligand binding positions^6^. Chemical probe methods might complement other methods for analyzing target molecule–ligand complex structures, such as NMR, X-ray crystallography, and cryo-electron microscopy, especially when the interactions are weak or thermally unstable.

Many types of mass spectrometry (MS) analyses for biomacromolecules are currently available, such as electrospray ionization (ESI)^7^ and matrix-assisted laser desorption/ionization (MALDI)^8–10^. These methods are highly sensitive, and PMF is applicable for µg~ng amount of proteins. Meanwhile, despite its lower sensitivity than the above “soft” ionization methods, LDI MS without matrixes has also been developed for the selective detection of molecular ions of small molecules that are directly irradiated by UV laser. For example, detection of the molecular ions of dipeptides having aromatic amino acids^8^ and polycyclic aromatic hydrocarbons (PAHs) including anthracene and pyrene^11–14^, have been reported. Recently, label-assisted laser desorption/ionization (LA-LDI) MS was reported as a method using pyrene as a detection tag by Kozmin and co-workers^15^. Since then, several related LDI MS methods have been reported, which used PAHs or rhodamine fluorophores^16–19^.

**Figure 1:**
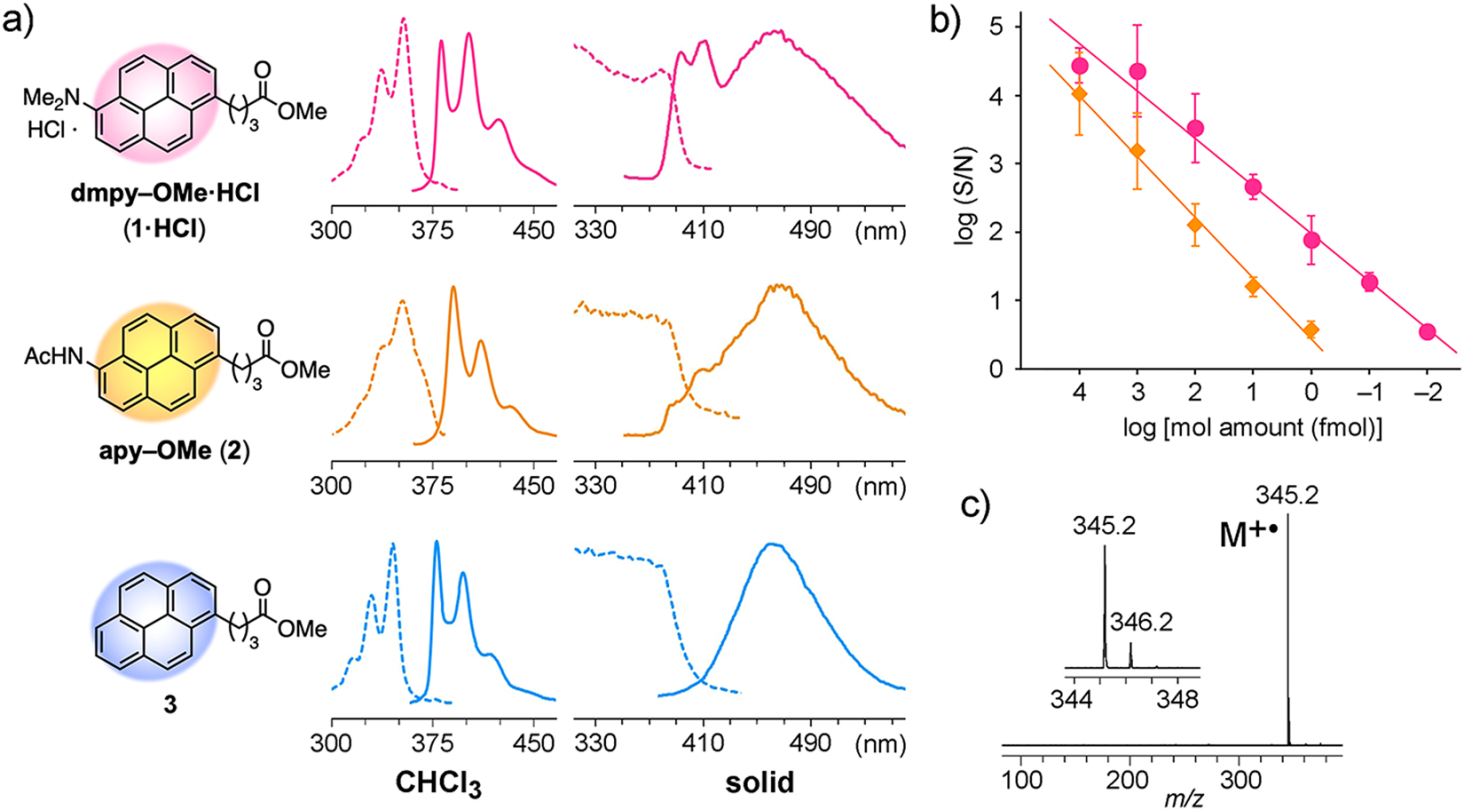
Discovery of the dmpy tag for LDI MS. (**a**) Structures and fluorescence spectra of pyrene derivatives. Excitation and emission spectra are shown as dashed and solid lines, respectively. (**b**) Sensitivity of detection of the molecular ion peaks ([M]^+•^) of **1** (circles) and **2** (diamonds) on LA-LDI MS. Values are the means ± SD of triplicate determinations. (**c**) LA-LDI mass spectrum of **1** (1 pmol).

We expected that LA-LDI MS could be applied to target–ligand interaction analysis if the labeled peptides are selectively detected from a mixture of unlabeled peptides. Several fluorescent dyes with cationic properties, such as 7-amino-4-methylcoumarine and Alexa Fluor 350, enhance the sensitivity of ESI and MALDI MS^20,21^. Thus, to improve the detection sensitivity of pyrene group, 6-aminopyrene was initially examined, but a homodimer (radical) cation was predominantly observed on LA-LDI MS, which was oxidatively dimerized *in situ* by UV laser irradiation^22^. Meanwhile, 6-amidopyrene methyl ester (apy–OMe, **2**) was highly detectable in amounts as low as 10 fmol by LA-LDI MS, and in some cases, apy-labeled peptides (from 200 pmol actin) were selectively detected^23^. However, 42 mu-reduced fragment ions that had lost ketene (CH_2_=C=O) were observed in association with the parent ions of apy tags. To inhibit such *in situ* fragmentations or oxidative dimerization on the pyrene tag and to further improve the sensitivity of detection for labeled peptides, we have newly developed *N*,*N*-dimethylaminopyrene (dmpy) as a highly efficient LDI-MS fluorescent tag (Fig. 1a). Here we describe LDI MS analysis of dmpy-tag and its application to the precise analysis of target molecule–ligand interactions.

## Results

### LA-LDI MS analysis and fluorescence spectra of dmpy tag

First, *N*,*N*-dimethylaminopyrene methyl ester (dmpy–OMe, **1**) was prepared from a 6-aminopyrene derivative by Borch reductive amination^24^ (Scheme S1). In LDI MS of **1**, a molecular ion peak at *m/z* 345.2 ([M]^+•^) was solely detected for an amount of only 0.1 fmol without any fragmentations, and its S/N (signal-to-noise) ratio was ca. 100 times higher than that of **2** (Fig. 1b,c). Since the samples for LDI MS are generally prepared by spotting a 50% aq. MeCN / 0.1% TFA solution and drying-up on a steel plate, **1** was protonated as a TFA salt, which might enhance ionization. In fact, the addition of weaker acids instead of TFA, such as acetic acid or formic acid, decreased the sensitivity of detection of dmpy tags on LA-LDI MS.

To evaluate the dmpy tag, spectroscopic properties were compared with those of other pyrene derivatives. The UV–VIS absorbance of **1** was almost identical to that of its hydrochloride salt (**1·HCl**) (Fig. S1), but shifted to a longer wavelength (λ_max_ 357 nm in CHCl_3_) compared to those of **2** and methyl 4-(1-pyrenyl)butyrate (**3**) (λ_max_ 346 and 340 nm)^22^, and almost matched the wavelength of the Nd:YAG laser (355 nm) on the LDI MS. As for the fluorescence spectra in CHCl_3_ solution, the Stokes shift of **1·HCl** (28 nm) was smaller than those of **2** (39 nm) and **3** (33 nm), while the excitation and emission spectrum patterns of the three compounds were similar (Fig. 1a, Table S2). Since LDI MS is performed by the laser irradiation of samples crystalized with or without matrixes, we compared the fluorescence spectra of pyrene derivatives in the solid state. All of the three pyrene derivatives **1·HCl**, **2**, and **3** had broad absorptions at <390 nm in their excitation spectra and maximum absorption at 462–465 nm in their emission spectra. However, only **1·HCl** had two additional strong emission peaks at shorter wavelengths (λ_em_ 396 and 413 nm). In addition, the fluorescence quantum yield of **1·HCl** in the solid state (Φ_F_ 0.068) was much lower than those of **2** and **3** (Φ_F_ 0.107 and 0.721, respectively). These results suggested that low fluorescence quantum yield and high heat emission property of the dmpy tag might cause effective heat transfer to improve the sensitivity of detection of **1** on LA-LDI MS.

### LDI MS and MS/MS analysis of dmpy-labeled peptides

**Figure 2:**
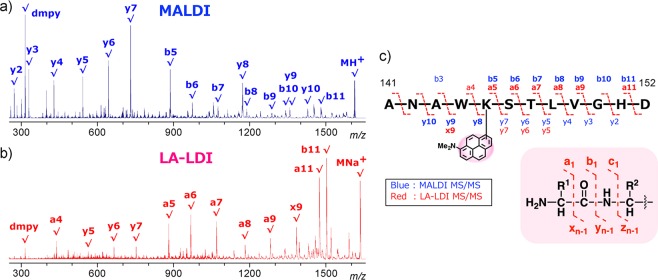
Comparison of the fragmentation patterns of dmpy-labeled peptide [ANAWK(dmpy)STLVGHD (1 nmol), *m/z* 1611.8 (M + H)^+^, 1633.8 (M + Na)^+^] on (**a**) MALDI and (**b**) LA-LDI MS/MS. (**c**) Assigned a-, b-, x-, and y-type fragment ions on MS/MS analyses.

To examine the LDI MS analysis, dmpy-labeled peptides were prepared by the condensation of Lys-containing *N*-Fmoc-peptides and dmpy *N*-hydroxysuccinate (dmpy–OSu, **4**) followed by treatment with piperidine. On LA-LDI MS, a molecular ion of dmpy-labeled hexapeptide [K(dmpy)ILTER] was detected with a S/N value of 95 using 150 pmol, while 2 nmol of the corresponding apy-labeled peptide was required for similar detection (Fig. S2). These minimum detectable amounts were much greater than those of **1** and **2**, because a- and c-type ions derived from in-source decay fragmentation were major peaks in both spectra. Notably, three a-type ions (a_2_, a_3_ and a_5_) were observed only in the dmpy-labeled peptide on LA-LDI MS. Due to the positively-charged dimethylamino group, fragments ions of the dmpy-labeled peptides might be efficiently detected than those of apy-labeled peptides. Meanwhile, in the MS/MS analysis of dmpy-labeled dodecapeptide [ANAWK(dmpy)STLVGHD], effective charge-remote fragmentation was observed on both MALDI and LA-LDI MS, but their fragmentation patterns were considerably different: b and y ions below *m/z* 900 were dominant on MALDI, while characteristic a and x ions were observed above the half molecular weight (*m/z* 800–1600) on LA-LDI (Fig. 2). Thus, dmpy tag was highly useful for complementary peptide sequence analysis by LDI MS.

### Use of dmpy as a fluorescent affinity tag

**Figure 3:**
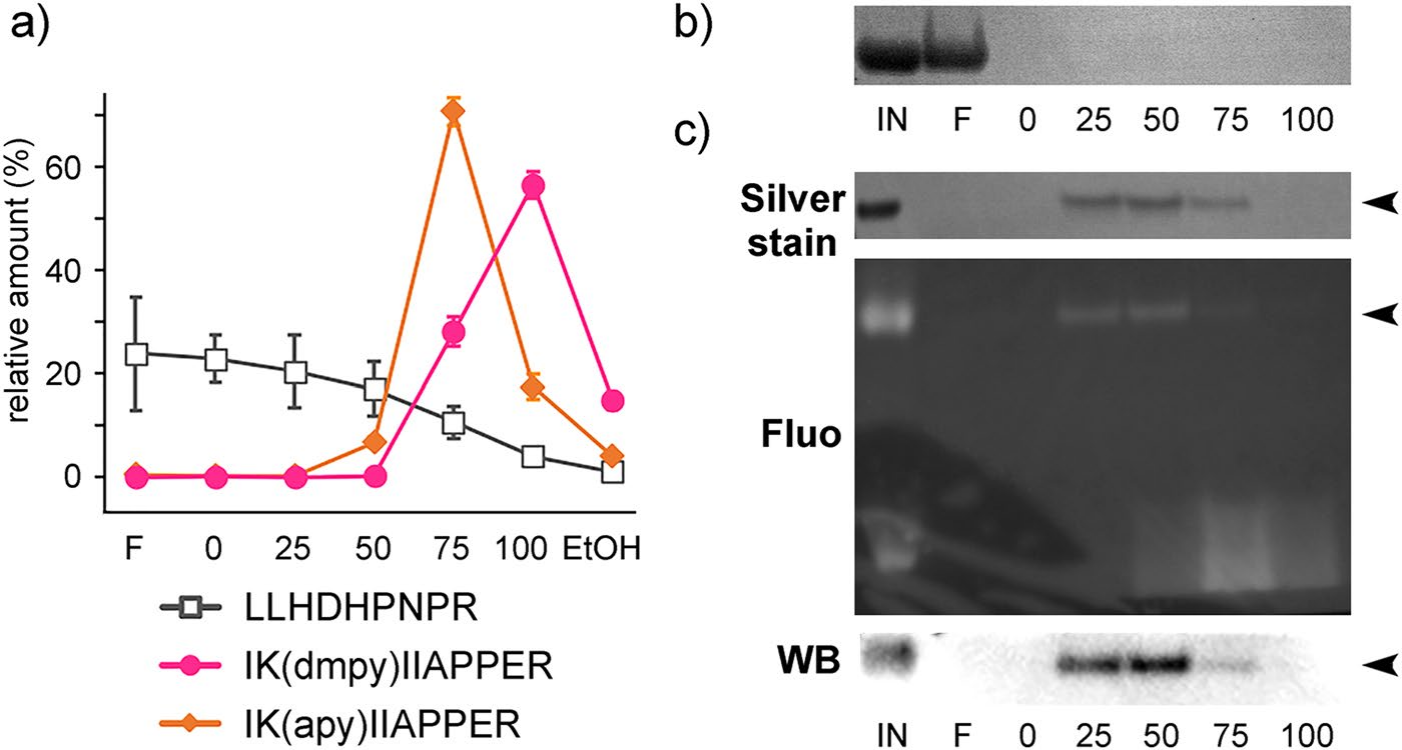
Affinity purification of dmpy-labeled peptides and proteins using the TSK-G3000S polystyrene gel. (**a**) Relative amounts of non-labeled and dmpy/apy-labeled peptides in each eluted aq. MeOH (0–100%) followed by EtOH fraction. Values are the means ± SD of triplicate determinations. The sequences of the peptides used are shown at the bottom. (**b**) Unlabeled avidin (Coomassie brilliant blue stain) was dominantly eluted in the flow-through (F) fraction. (**c**) dmpy-labeled avidin (arrowheads) was eluted stepwise with aq. EtOH (0–100%). Labeled avidin was detected by silver stain, dmpy fluorescence, and immunoblotting analysis using anti-benzo[a]pyrene (BAP) antibody.

In general, it is essential to remove high-concentration nonvolatile salts (such as guanidine) from the sample solution to improve the sensitivity of both MALDI and LA-LDI MS. Previously, we demonstrated that a TSK-G3000S gel (styrene-divinylbenzene co-polymer) was useful for the desalting of apy-labeled peptides^25^, as well as for the purification of water-soluble natural products^26–28^. Since **1** has a larger cLogP value (5.631) than those of **2** (4.485) and **3** (5.466), we expected that dmpy-labeled peptides might be more hydrophobic and interact with this gel more strongly than apy derivatives. With this gel, a non-labeled nonapeptide [LLHDHPNPR] was eluted between the 0 and 75% aq. MeOH fractions, and an apy-labeled nonapeptide [IK(apy)IIAPPER] was mostly in the 75% aq. MeOH fraction. In contrast, a dmpy-labeled nonapeptide [IK(dmpy)IIAPPER] was specifically eluted in the 100% aq. MeOH fraction, with almost complete separation from non-labeled peptides (Figs. 3a and S3). As for proteins, dmpy-labeled avidin and remaining small dmpy reagents were mostly eluted in the 25–50% and 75% aq. EtOH fractions, respectively, while native avidin was hardly absorbed on this gel (Fig. 3b,c). Similar to an octadecylsilyl (ODS) resin, the elution power of aq. EtOH on a TSK-G3000S polystyrene gel was greater than that of aq. MeOH. Furthermore, dmpy-labeled proteins, but not apy-labeled ones, were specifically detected with anti-benzo[a]pyrene antibody (Figs. 3c and S4). Thus, dmpy was found to be an excellent affinity fluorescent tag for the purification of peptides and proteins.

### Development of a ligand-dissociation-type dmpy probe

**Figure 4:**
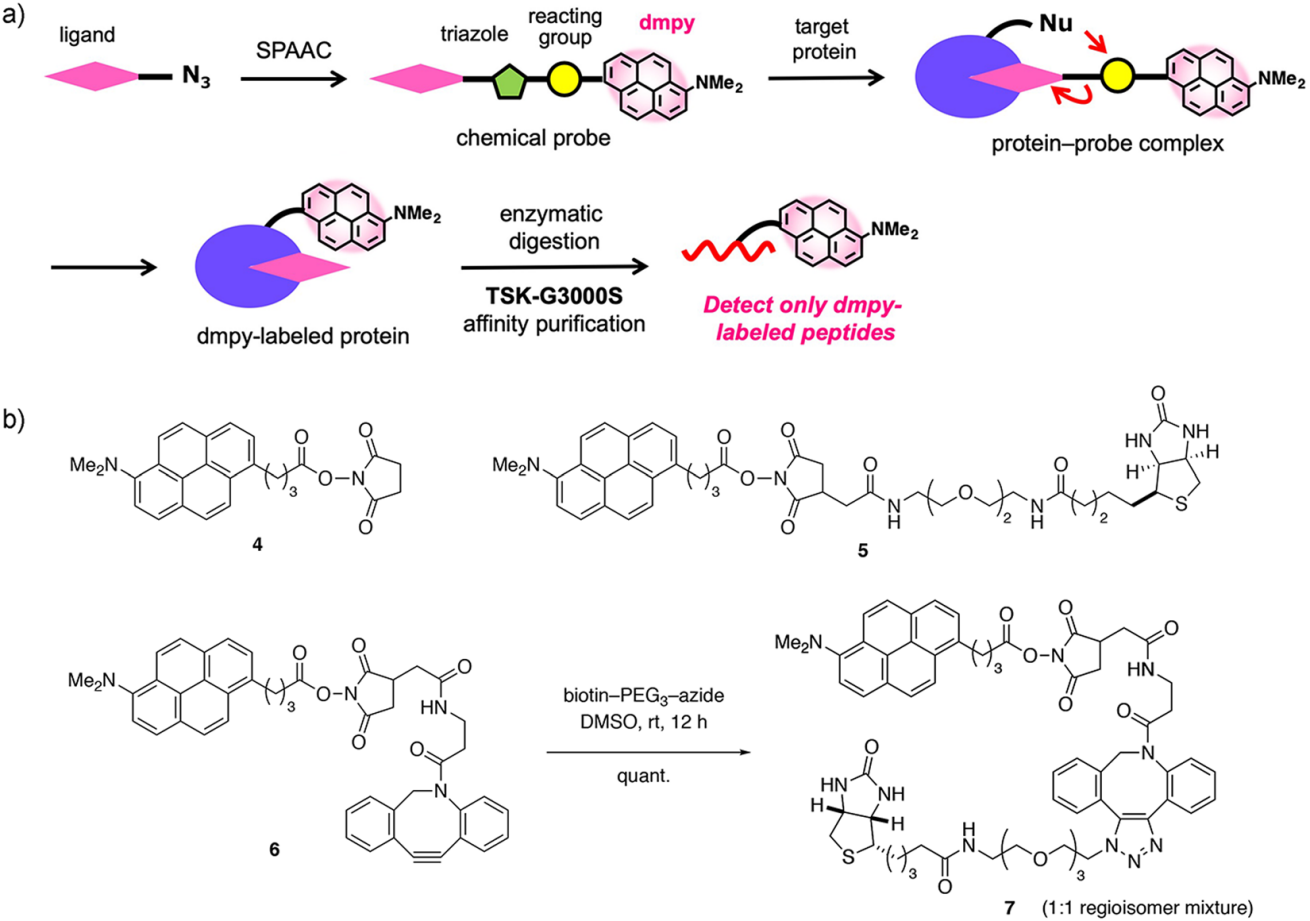
Design and synthesis of dmpy probes. (**a**) Overall strategy for the binding-position analysis of target proteins with ligand-dissociation-type dmpy probes. (**b**) Structures of dmpy probes **4** and **5**, and the synthesis of dmpy biotin probe **7** by SPAAC reaction.

We have developed apy biotin probes having an *N*-hydroxysuccinimidyl (NHS) ester to utilize in PMF analysis and covalent docking simulation^25^. Among them, a ligand-dissociation-type apy biotin probe (apy–OSu–biotin) selectively labeled K135 of avidin, which is closest to the biotin-binding site among the eight Lys residues in the avidin–biotin complex (PDB: 2avi). Thus, by using ligand-dissociation-type dmpy probes, the covalent bonds between the NHS groups and the ligands are cleaved by the attack from target molecules, and only the dmpy group might covalently bind to the near ligand-binding position of protein, as with previous apy probe studies (Fig. 4a). To demonstrate this strategy, dmpy–OSu–biotin (**5**) was prepared (Fig. 4b, Scheme S1).

In addition, to develop more versatile and practical methods to analyze the binding mode of target proteins, we attempted to use strain-promoted azide–alkyne cyclization (SPAAC)^29,30^ for new dmpy probes. Thus, dmpy–OSu–DBCO (**6**) was synthesized, where the reactive NHS ester was placed between the dmpy and dibenzocyclooctyne (DBCO) moieties. Subsequent SPAAC reaction of **6** with a PEG-linked biotin azide in DMSO solution afforded dmpy biotin probe **7** in quantitative yield (as an ~1:1 mixture with the regioisomer **7’** at the triazole moiety), which was directly used for the protein-labeling experiments.

### Binding-position analysis of avidin using a dmpy biotin probe

With dmpy biotin probes in hand, avidin was labeled with **4** (20 eq.), **5** (1 eq.) or **7** (3 eq.) in 2 mM sodium bicarbonate/phosphate buffered saline (PBS). MALDI MS of the avidin protein labeled with **4** showed that the major products were the adducts of 4–10 dmpy molecules (Fig. S5). In contrast, with biotin probes **5** and **7**, the adduct of only one dmpy molecule was observed with native avidin (~70% and ~30% conversions based on the mass intensity, respectively). Thus, both of these two biotin probes reacted with avidin as the 1:1 ratio.

**Figure 5:**
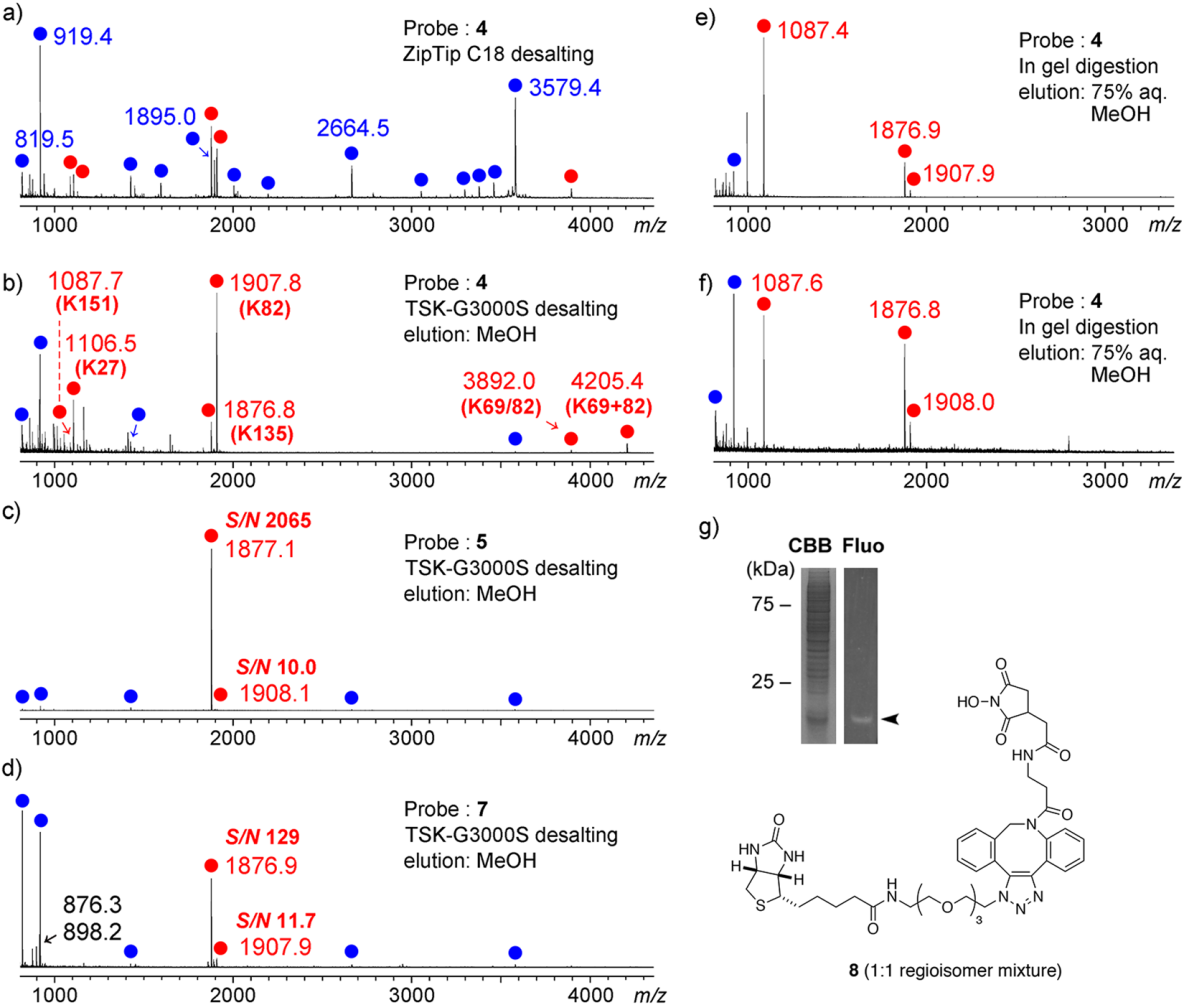
MALDI MS of the tryptic peptides of avidin (10 pmol) labeled with dmpy probes **4**, **5**, and **7**. Red and blue circles indicate dmpy-labeled and unlabeled peptides, respectively. In (**a**), labeled peptides after tryptic digestion were desalted with ZipTip C_18_ and eluted with a matrix solution for MALDI MS. In (**b**–**d**), labeled peptides were desalted with TSK-G3000S gel and eluted stepwise with aq. MeOH. In (**e**), dmpy-labeled avidin was loaded on the TSK-G3000S gel and then digested with trypsin. dmpy-labeled peptides were eluted stepwise with 75% aq. MeOH. In (**f**), a mixture of HEK293 cell lysate and dmpy-labeled avidin (10:1 w/w protein) was loaded on the TSK-G3000S gel. In-gel digestion and selective elution of dmpy-labeled peptides was performed as in (**e**). (**g**) SDS-PAGE analysis of the sample used in (**f**). Arrowhead indicates dmpy-labeled avidin.

To determine the dmpy-labeled positions, avidins reacted with three probes were denatured with 6 M guanidine·HCl. Subsequent reductive alkylation with DTT and iodoacetamide, and trypsin digestion provided dmpy-labeled avidin peptides. To remove high concentration salts from the peptide mixture samples, a conventional reversed-phase ODS tip column (ZipTip C18) was initially used, but the relative intensities of dmpy-labeled peptides on MALDI MS were fairly low compared to those of non-labeled peptides (Fig. 5a). In contrast, when we used a TSK-G3000S gel batch column, the relative intensities of dmpy-labeled peptides were highly increased (Fig. 5b). As for non-specific labeling with **4**, six dmpy-labeled peptides were detected in the 100% MeOH fraction, where the NHS ester in **4** reacted with the Lys ε-amino groups (K27, K69, K82, K135, or K151) (Table S1).

In the case of ligand-dissociation-type probe **5**, a dmpy-labeled peptide (No. 6, *m/z* 1877.1) was exclusively observed, and its sequence was S^125^SVNDIGDDWK^135^(dmpy)ATR^138^, as determined by MS/MS analysis (Figs. 5c and S6). Despite the presence of a structurally bulky diphenylazacyclooctene group, avidin-labeling by another ligand-dissociation-type dmpy probe **7** was also highly specific, comparable to probe **5**, and the relative intensity of the largest dmpy-labeled peptide (*m/z* 1876.9, same as **5**) was >10 times higher than the second largest one (No. 7, *m/z* 1907.9) (Fig. 5d). Along with dmpy-labeled peptides, two non-labeled small peptides (Nos. 3 and 4, *m/z* 819.5 and 919.4) were observed as major peaks, which had tryptophan or phenylalanine residues. In addition, *N*-hydroxyimide **8**, a hydrolyzed product of probe **7**, was detected [*m/z* 876.3 (M + H)^+^ and 898.2 (M + Na)^+^]. These aromatic compounds might have some hydrophobic interactions with the polystyrene gel, as with the dmpy tag.

**Figure 6:**
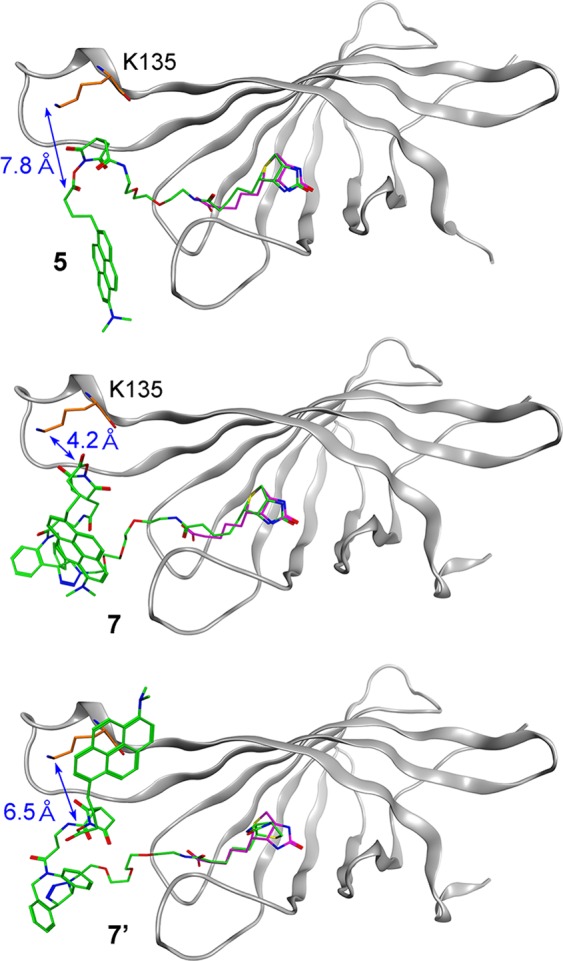
Docking simulations of dmpy biotin probes **5**, **7**, and **7’** with avidin. The most stable conformers of probes on avidin are shown in green. The ligand biotin on the original avidin–biotin complex (PDB: 2avi) and the K135 residue are highlighted in magenta and orange, respectively.

Labeling specificity by probes **5** and **7** was evaluated by molecular modeling studies, where the biotin pocket was settled as a ligand-binding site (Fig. 6). All of the NHS ester groups in **5**, **7**, and **7’** (a regioisomer of **7** at the triazole moiety) were closest to the K135 ε-amino group (7.8, 4.2, and 6.5 Å) in the most stable conformers, and too far away to bind to other Lys residues (18.9~46.9 Å) (Table S3). The S values (ligand-binding affinities for the free energy) of the most stable conformers of **5**, **7**, and **7’** were –10.3387, –11.2929, and –11.5532 kcal/mol, respectively. All of the biotin moieties of these probes well-overlapped that in the original avidin–biotin complex, with the RMSD values of the 15 biotin atoms (C, N, O, and S) of 0.58, 0.83 and 2.49 Å, respectively. Thus, the specific labeling of K135 residue by dmpy biotin probes **5** and **7** was reliable, as in the case of the apy–OSu–biotin probe^25^.

### Affinity purification and in-gel digestion of dmpy-labeled proteins

To more selectively detect dmpy-labeled peptides on LDI-MS, we also examined in-gel digestion using the TSK-G3000S gel. The avidin labeled with **4** was denatured with 6 M guanidine·HCl at 95 °C, and loaded on the gel. After the removal of high-concentration guanidine salt and unreacted avidin, dmpy-labeled avidin was *S*-alkylated and digested with trypsin, and the resultant dmpy-labeled peptides were eluted stepwise with aq. MeOH. As a result, three highly enriched dmpy-labeled peptides (No. 1, *m/z* 1087.4, and Nos. 6 and 7) were detected in the 75% aq. MeOH fraction (Fig. 5e). The sequence of the major peptide (No. 1) was L^147^TQK^151^(dmpy)E^152^ (Fig. S6), which was located at the C-terminus of avidin. While the avidin labeled with **4** had 4–10 dmpy groups, the dmpy tag at the K^151^ residue might strongly bind to the polystyrene gel.

Furthermore, to show the utility of the dmpy group as an affinity tag, we tried to purify dmpy-labeled peptides from a mixture of dmpy-labeled avidin and the HEK293 cell lysate. The protein mixture precipitated with acetone was absorbed on the TSK-G3000S gel, and in-gel digestion and selective elution were similarly performed as mentioned above. The same three dmpy-labeled peptides (Nos. 1, 6, 7) as above were dominantly eluted in the 75% aq. MeOH fraction (Fig. 5f,g), and no major peaks derived from the cell lysate proteins were observed.

## Discussion

To readily determine the binding mode of target protein–ligand interactions, dmpy, a versatile fluorescent affinity mass tag, has been developed. This tag was detected at an amount of 0.1 fmol by LA-LDI MS, and was useful for peptide MS/MS analysis via effective charge-remote fragmentation. So far, we have aimed at solely detecting pyrene-labeled peptide(s) from a mixture of tryptic peptides by LA-LDI MS^22,23^. However, the detectable sensitivity of dmpy-labeled peptides was not improved, due to the in-source decay fragmentation. Meanwhile, we found that dmpy-labeled peptides were more hydrophobic and strongly interacted with a TSK-G3000S polystyrene gel than apy-labeled or non-labeled peptides. Thus, now we performed affinity purification of dmpy-labeled molecules.

We demonstrated that ligand-dissociation-type dmpy probes, prepared in a single step from azide-conjugated ligands, specifically reacted at the ligand-binding sites of target proteins. In combination with tryptic digestion and the TSK-G3000S polystyrene gel affinity purification, an expected dmpy-labeled peptide was predominantly detected by MALDI MS. This polystyrene gel was also useful for the desalting and affinity purification of dmpy-labeled proteins, which were specifically detected by anti-benzo[a]pyrene antibody. These results suggest that combination of the dmpy probe-labeling method and affinity purification with a polystyrene gel enables us to directly identify the target proteins of bioactive ligands from the mixture of proteins (such as the cell lysate and the cell surface environment), and to precisely analyze their binding positions at once. Furthermore, by replacing from the NHS esters to other reacting groups, our chemical probe method might be applicable to many kinds of proteins that do not have a reactive Lys residue around their ligand-binding sites, and to other hydrophilic biomacromolecules, such as nucleotides and glycans.

From the viewpoint of the chemical labeling of target proteins, photoaffinity labeling is one of the most powerful tools^4,31–33^. Previous study showed that diazirine groups^34,35^ retained their photoreacting activities even in the presence of fluorescent pyrene or nitrogen-substituted pyrene groups^22,23^. Thus, our dmpy tag could be utilized in combination with various common reacting groups to develop activity-based chemical probes. Further studies on chemical labeling and selective detection with the use of dmpy probes for various target biomacromolecules including membrane-bound proteins or labile protein–protein complexes are currently underway.

## Methods

### LDI-MS analysis

Matrix-assisted laser desorption/ionization with time-of-flight mass spectrometry (MALDI-TOF MS) was performed using a Bruker UltrafleXtreme or Ultraflex III spectrometer equipped with a 355 nm Nd:YAG laser (Smartbeam 1000 or 200 MHz), with α-cyano-4-hydroxycinnamic acid (α-CHCA) or sinapinic acid as matrices^22^. Label-assisted laser desorption/ionization mass spectrometry (LA-LDI MS) was performed using a Bruker UltrafleXtreme spectrometer as for MALDI-TOF MS without matrices^25^. Samples dissolved in 50% aq. MeOH or MeCN / 0.1–1% TFA were spotted on an MTP384 ground steel target plate and air-dried according to the manufacturer’s instructions. To determine the detectable sensitivity of the molecular ion peaks of dmpy–OMe (**1**) and apy–OMe (**2**) on LA-LDI MS, standard parameters for the reflector positive mode of MALDI MS were used with 20% laser intensity (Fig. 1b,c). For the LA-LDI MS and MS/MS analyses of dmpy-labeled peptides (Figs. 2 and S2), standard parameters for the reflector positive mode of MALDI MS were used except for detector gain voltage (changed from 1650 to 1860 V). Tandem MALDI MS/MS analysis was performed using a Bruker UltrafleXtreme spectrometer (Fig. S6).

### Unspecific protein-labeling using dmpy probe 4 and their detection

To a solution of avidin (85 µg, 5 nmol) or streptavidin (65 µg, 5 nmol) in phosphate-buffered saline (PBS) and 2 mM NaHCO_3_ (240 µL) was added 10 mM dmpy–OSu (**4**) in DMSO (10 µL, 100 nmol), 10 mM apy–OSu in DMSO^23^ (10 µL, 100 nmol), or DMSO (10 µL), and the resulting mixture was stand for 48 h at room temperature. The resulting solution containing dmpy-labeled proteins (5 µL) was desalted using a pipette-attached tip column (Zip-Tip C_4_, Millipore) for protein MS analysis (Fig. S5), as mentioned previously^25^. An aliquot of labeled samples (10 µL) were mixed with 2 × SDS buffer (10 µL, Sigma), and boiled at 95 °C for 5 min. SDS-PAGE was performed with a 15% precast polyacrylamide gel (ATTO). After the fixation of proteins in AcOH/MeOH/H_2_O (1:4:5) for 30 min at room temperature and the washings with water for 5 min (for three times), labeled proteins were detected with a fluorescent imaging analyzer (ATTO, WSE-5200 Printgraph 2 M, excitation UV 365 nm), followed by CBB-staining using a Quick-CBB kit (Wako) or silver staining using a Silver Stain Kit for Protein (GE Healthcare) (Figs. 3 and Fig. S4). For immunoblot analysis, proteins in the gels after electrophoresis were transferred to PVDF membranes, using the Trans-Blot SD semi-dry blotting system (Bio-Rad), according to the manufacturer’s instructions. Labeled proteins were treated with mouse monoclonal anti-benzo[a]pyrene (BAP-13) (1:50~1:100, cat. no. sc51508, Santa Cruz Biotechnology) and then with HRP-conjugated anti-mouse IgG (1:4000, cat. no. NA931, GE Healthcare). The HRP-conjugated bands were visualized with an ECL-prime system (GE Healthcare), and detected by a Fujifilm LAS-4000 mini imaging scanner.

### Labeling of avidin with dmpy biotin probes and trypsin digestion

To a solution of avidin (85 µg, 5 nmol) in PBS and 2 mM NaHCO_3_ (240 µL) was added 1.5 mM dmpy biotin probes **5** or **7** in DMSO (10 µL, 15 nmol), and the resulting mixture was stand for 48 h at room temperature, as described previously^23,25^. The resulting solution was lyophilized, dissolved in 6 M guanidine·HCl in 50 mM NH_4_HCO_3_ (60 µL), and heated at 95 °C for 15 min. To the resulting denatured protein was added 45 mM DTT in 50 mM NH_4_HCO_3_ aq. (4 μL), and the solution was incubated at 50 °C for 15 min. The solution was then reacted with 100 mM iodoacetamide in 50 mM NH_4_HCO_3_ aq. (4 μL) at room temperature for 15 min. After the resulting solution was diluted with 50 mM NH_4_HCO_3_ (512 µL) (to avoid deactivation of trypsin at a high guanidine concentration), sequence-grade modified trypsin (100 ng/μL, #V5111, Promega) in acetate buffer (20 μL) was added, and the resulting mixture was incubated at 37 °C for 20 h. Hydrolysis was stopped by the addition of 10% TFA aq. (15 μL). The avidin reacted with an excess amount of dmpy–OSu (**4**) as mentioned above was similarly digested with trypsin.

### Affinity purification of dmpy-labeled peptides and proteins

Desalting of tryptic peptides with the use of gel-permeation resin was performed as follows^25^: 1/20 amount (31 µL) of the above tryptic peptide solutions were diluted with water (69 µL), and loaded on a TSK-G3000S polystyrene gel (Tosoh Co., 10 µL, equilibrated with water). After the filtrates were removed using centrifuge filter units, the resin was washed with 0%, 25%, 50%, 75%, and 100% aq. MeOH (in order) (100 µL each). The 1/25 amounts of eluate (4 µL, 10 pmol based on the original avidin protein) were used for MALDI MS analysis (Fig. 5b–d). The sequences of dmpy-labeled peptides were established by MALDI MS/MS analysis (Fig. S6). For comparison, 1/500 amount (1.2 µL) of the above tryptic peptide solution was used for desalting using a pipette-attached tip column (Zip-Tip C_18_, Millipore), and eluted with α-CHCA in 50% aq. MeCN/0.1% TFA for MALDI MS analysis (Fig. 5a).

For the synthetic dmpy- or apy-labeled peptides, mixtures of a non-labeled peptide (LLHDHPNPR, 1 nmol) and a dmpy-labeled peptide [IK(dmpy)IIAPPER, 250 pmol] or an apy-labeled peptide [IK(apy)IIAPPER, 750 pmol] in 50 µL water was loaded on the TSK-G3000S gel (10 µL). After the filtrate was removed, the resin was washed with 0%, 25%, 50%, 75%, and 100% aq. MeOH [and EtOH, optional] (50 µL each). The 1/25 amounts of eluate (total 40, 10, and 30 pmol for non-labeled, dmpy-labeled, and apy-labeled peptides, respectively) were analyzed by MALDI MS (Fig. S3). Also, relative amounts of labeled and unlabeled peptide samples were determined by the analytical HPLC based on the UV (280 nm) and fluorescence (λ_ex/em_ 365/407 nm) (Fig. 3a).

For the dmpy-labeled proteins, a solution of the avidin reacted with dmpy–OSu (**4**) (20 µL, 400 pmol, ca. 7 µg protein) was diluted with water (80 µL), and loaded on the TSK-G3000S gel (10 µL). After the filtrate was removed, the resin was washed with 0%, 25%, 50%, 75%, and 100% aq. EtOH (in order) (100 µL each). The flow-through fraction was desalted by Amicon Ultra YM-10 (MWCO 10 kDa, Merck Millipore), and all eluates were freeze-dried and analyzed by SDS-PAGE (Fig. 3b,c).

### In gel digestion using the TSK-G3000S polystyrene gel

A solution of the avidin reacted with dmpy–OSu (**4**) (15 µL, 300 pmol) was mixed with 6 M guanidine·HCl in 50 mM NH_4_HCO_3_ (75 µL), and heated at 95 °C for 15 min. The resulting denatured protein was diluted with 25 mM NH_4_HCO_3_ (162 µL) and mixed with the TSK-G3000S gel (10 µL). After the filtrate was removed, the resin was washed with 25 mM NH_4_HCO_3_ (100 µL, the same buffer was used for the following steps), and incubated with 10 mM DTT in 25 mM NH_4_HCO_3_ aq. (100 μL) at 56 °C for 45 min. After the removal of the filtrate, the resin was washed and incubated with 55 mM iodoacetamide in 25 mM NH_4_HCO_3_ aq. (100 μL) at room temperature for 30 min. After the removal of the filtrate, the resin was washed and incubated with sequence-grade modified trypsin (20 ng/μL) in 25 mM NH_4_HCO_3_ aq. (100 μL) at 37 °C for 20 h. After the filtrates were removed, the resin was washed with 0%, 25%, 50%, 75%, and 100% aq. MeOH (in order) (100 µL each). The 1/30 amounts of eluate (3.3 µL, 10 pmol based on the original avidin protein) were analyzed by MALDI MS (Fig. 5e).

For the affinity purification of dmpy-labeled avidin from the mixture of cell lysate proteins, human embryonic kidney 293 (HEK293) cells were cultured in Dulbecco’s Modified Eagle’s Medium (DMEM) supplemented with fetal bovine serum (FBS, 10%) in a humidified atmosphere containing CO_2_ (5%). The cells were cultivated on a six-well plate until they reached 90% confluence, washed twice with cold PBS, scraped, and collected by centrifugation. The cells were lysed in 30 µL of RIPA buffer with 10% protease inhibitor cocktail (100×, Nacalai tesque). After suspending by voltex (10 sec × 3) and standing on ice for 30 min, the suspension was centrifuged (15,000 rpm, 4 °C, 30 min) to give cell lysate (13 mg protein/mL). The protein concentration was measured with a TaKaRa BCA Protein Assay Kit with BSA as a standard. A solution of avidin reacted with **4** (15 µL, 300 pmol, ca. 5 µg protein) was mixed with the cell lysate (50 µg protein) in RIPA buffer (15 µL), and then treated with cold (−20 °C) acetone (90 µL). After standing at −20 °C for 3.5 h, the suspension was centrifuged (12,000 × *g*, 4 °C, 5 min) to give the precipitate, which was denatured with 6 M guanidine·HCl in 50 mM NH_4_HCO_3_ (30 µL) at 95 °C for 15 min. In gel digestion using TSK-G3000S gel, and the detection of dmpy-labeled peptides by MALDI MS was performed similarly as mentioned above (Fig. 5f).

### Molecular modeling

Molecular modeling studies of the avidin–probe complexes were performed using the Molecular Operating Environment (MOE) 2018.0101 program package (Chemical Computing Group, Inc.), similarly as mentioned previously^25^. For docking model studies, all water molecules in the avidin–biotin complex (PDB: 2AVI) were removed except water molecules around the ligand, and all protons on the proteins and ligands in the complex were complemented. A conformational search was performed using the Amber14:EHT force-field with GB/VI Generalized Born^35^ implicit solvent electrostatics (*D*_in_ = 1, *D*_out_ = 80) and with LowModeMD^36^. In the docking simulations of dmpy–biotin probes with avidin, the biotin pocket generated by the Site-Finder mode was settled as the ligand-binding site, which included 23 residues (N36, L38, S40, Y57, T59, V61–T64, W94, F96–S99, T101, F103, W121, L123–S126, R138, and N142). All the remaining probe parts were freely flexible on the target site (docking box).

## Supplementary information

Supplementary information.

## Data Availability

For the details of the synthetic procedures, ^1^H and ^13^C NMR spectra, and mass spectra of the compounds, and HPLC charts of synthetic peptides in this manuscript, see Supplementary Methods.
