# Supplementary material for: N,N-Dimethylaminopyrene as a fluorescent affinity mass tag for ligand-binding mode analysis
Source: Sci Rep. 2020 Apr 30;10:7311. doi: 10.1038/s41598-020-64321-9 (PMC7192892; doi:10.1038/s41598-020-64321-9)
Supplement: Supplementary file 1 — Supplementary information. [file 41598_2020_64321_MOESM1_ESM.pdf]

Supporting Information for

N,N-Dimethylaminopyrene as a fluorescent affinity mass tag for ligand-binding mode analysis

Atsushi Arai,[1] Rei Watanabe,[2] Atsunori Hattori,[1] Keita Iio,[2] Yaping Hu,[2] Kozo Yoneda,[2] Hideo Kigoshi [2,*] and Masaki Kita [1,2,*]

[1] Graduate School of Bioagricultural Sciences, Nagoya University, Furo-cho, Chikusa, Nagoya 464-8601, Japan. [2] Graduate School of Pure and Applied Sciences, University of Tsukuba, 1-1-1 Tennodai, Tsukuba 305-8571, Japan. Correspondence and requests for materials should be addressed to M. K. (email: mkita@agr.nagoya-u.ac.jp) and H. K. (email: kigoshi@chem.tsukuba.ac.jp)

(25 pages)

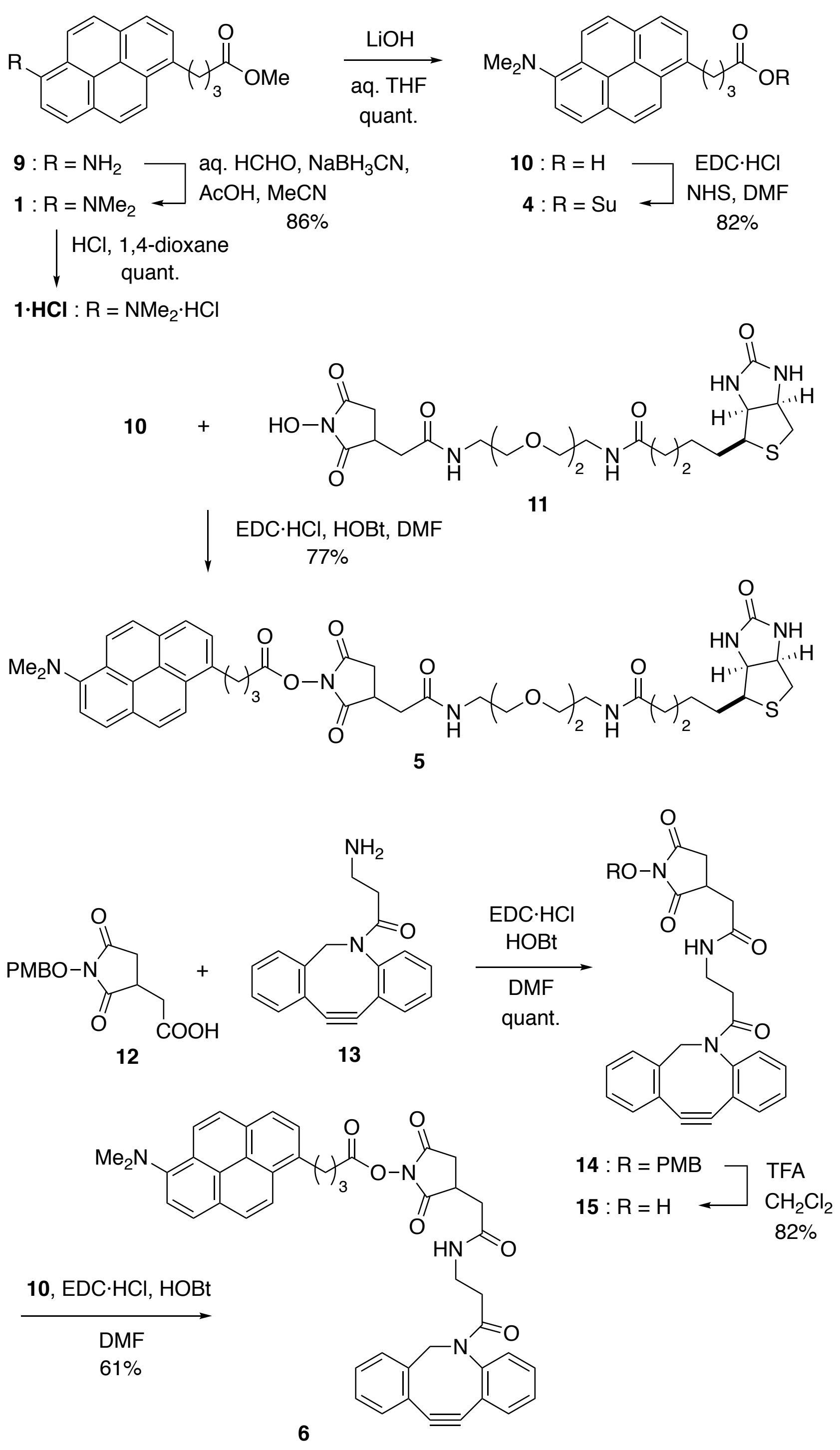

Scheme S1. Synthesis of dmpy NHS esters **4**–**6**.

Table S1. Tryptic peptides of the avidin labeled with dmpy probes **4**, **5**, and **7** detected by MALDI MS.

No.	Observed (*m/z*) [a]						Calculated (*m/z*) [a]		Start/end	Sequence [b]
	(Fig. 5a)	(Fig. 5b)	(Fig. 5c)	(Fig. 5d)	(Fig. 5e)	(Fig. 5f)	dmpy peptide	peptide		
1	774.5<br>**1087.7**	**1087.7**			**1087.4**	**1087.6**	**1087.6**	774.4	147 / 152	LRTQ**K**E
2	**1106.7**	**1106.5**					**1106.5**	793.4	27 / 33	**K**CSLTGK
3	819.5	819.5	819.2	819.3		819.5		819.5	119 / 124	TMWLLR
4	919.4	919.4	919.4	919.4	919.5	919.6		919.5	139 / 146	VGINIFTR
5	1425.8	1425.5	1425.8	1425.6				1425.7	84 / 95	TQPTFGFTVNWK
6	**1877.0**	**1876.8**	**1877.1**	**1876.9**	**1876.9**	**1876.8**	**1876.9**	1563.7	125 / 138	SSVNDIGDDW**K**ATR
7	1595.0<br>**1908.1**	**1907.8**	**1908.1**	**1907.9**	**1907.9**	**1908.0**	**1908.0**	1594.8	70 / 83	ESPLHGTQNTIN**K**R
8	1895.0							1894.9	96 / 111	FSESTTVFTGQCFIDR
9	2003.1							2003.0	51 / 69	GEFTGTYITAVTATSNEIK
10	2195.2							2194.0	96 / 114	FSESTTVFTGQCFIDRNGK
11	2664.5		2664.3	2664.4				2663.3	96 / 118	FSESTTVFTGQCFIDRNGKEVLK
12	3052.7<br>3296.9<br>3376.9							1835.9 [c]	34 / 50	WTNDLGS<u>N</u>MTIGAVNSR
13	3459.0							3457.7	83 / 111	RTQPTFGFTVNWKFSESTTVFTGQCFIDR
14	3579.4<br>**3892.8**	3579.4<br>**3892.0**<br>**4205.4**	3578.2	3578.9			**3891.9**<br>**4205.3**	3578.8	51 / 83	GEFTGTYITAVTATSNEI**K**ESPLHGTQNTIN**K**R

[a] The data represent the monoisotopic ion peaks $(M+H)^+$ values. Bold value means the data for dmpy-labeled peptides.

[b] "**K**" means dmpy-labeled lysine residue. All cysteine residues are carbamidomethylated.

[c] Calculated value without *N*-glycans on the N41 residue (underlined). The common *N*-terminal sequences (W34–S40) were determined by MS/MS analysis.

Table S2. Fluorescent properties of pyrenes in solution and solid states.

	In $CHCl_3$				In solid		
	Excitation (nm)	Emission (nm)	Stokes shift (nm)	Φ_F	Excitation (nm)	Emission (nm)	Φ_F
dmpy–OMe (**1·HCl**)	338, 353	381, 401	28	0.118	381	396, 413, 465	0.068
apy–OMe (**2**)	351	390, 411	39	0.147	–	409, 465	0.107
pyrene **3**	330, 345	378, 398	33	0.063	–	462	0.721

Table S3. Calculated distances between the Lys residues on avidin and the succinyl ester carbons in dmpy biotin probes **5**, **7**, and **7'** in the most stable complex conformers obtained by docking simulations (see Figure 6).

Lys residue	Distance (Å)		
	**5**	**7**	**7'**
27	40.24	38.59	40.32
33	32.81	32.81	32.46
69	21.25	23.89	22.38
82	46.64	44.79	46.93
95	18.93	21.69	21.73
114	40.41	37.91	38.44
118	32.24	28.83	30.23
135	7.77	4.22	6.49
151	45.69	39.94	39.68

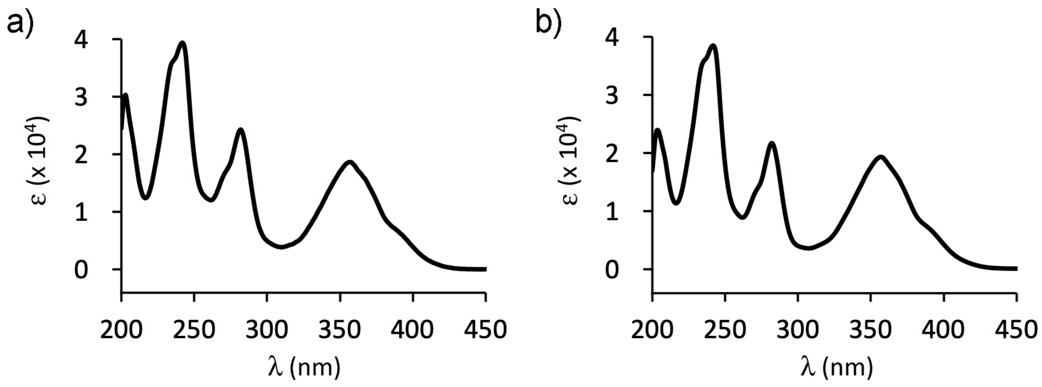

Figure S1. UV–Vis spectra of dmpy–OMe (**1**) and dmpy–OMe·HCl (**1·HCl**) in MeOH. Note that the λ_{max} and ε values of **1·HCl** were almost identical to **1**.

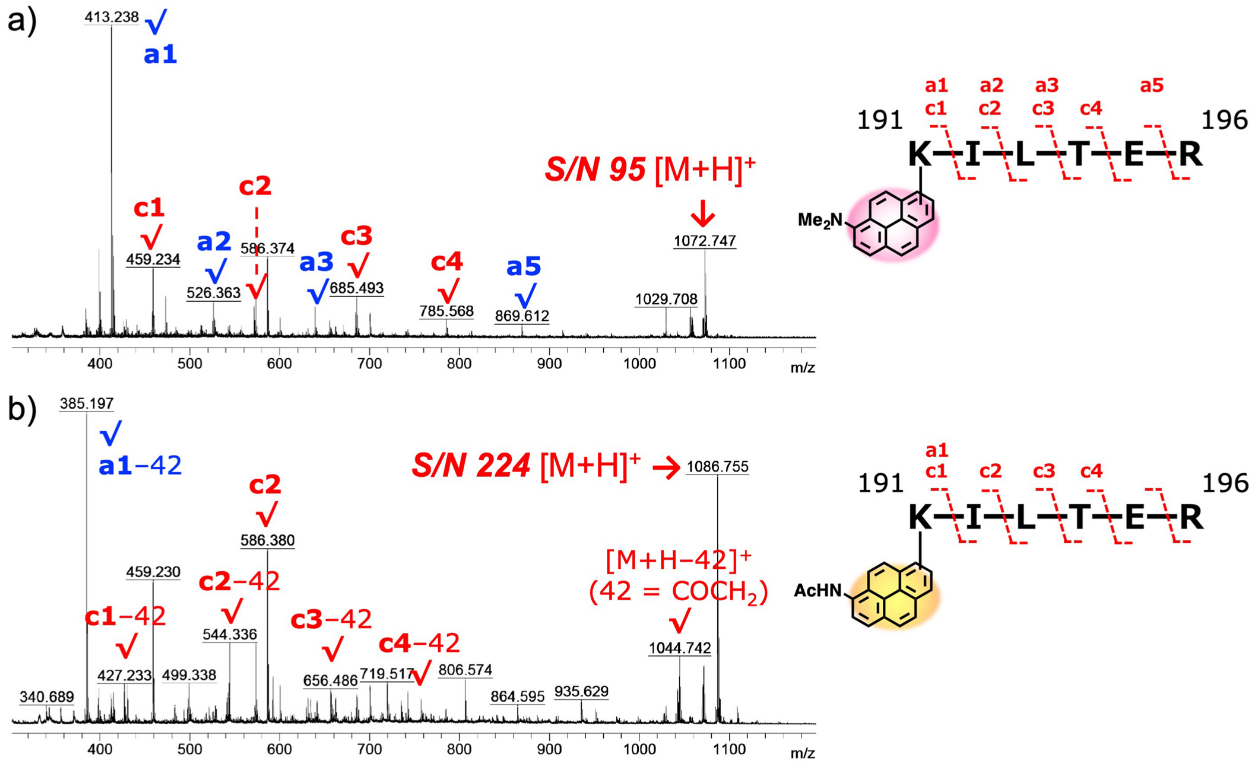

Figure S2. LA-LDI MS of dmpy/apy-labeled peptides. (a) K(dmpy)ILTER (150 pmol), *m/z* 1072.7 $(M+H)^+$. (b) K(apy)ILTER (2 nmol), *m/z* 1086.8 $(M+H)^+$. The a- and c-type fragment ions caused by the in-source decay on LDI MS are shown in blue and red, respectively.

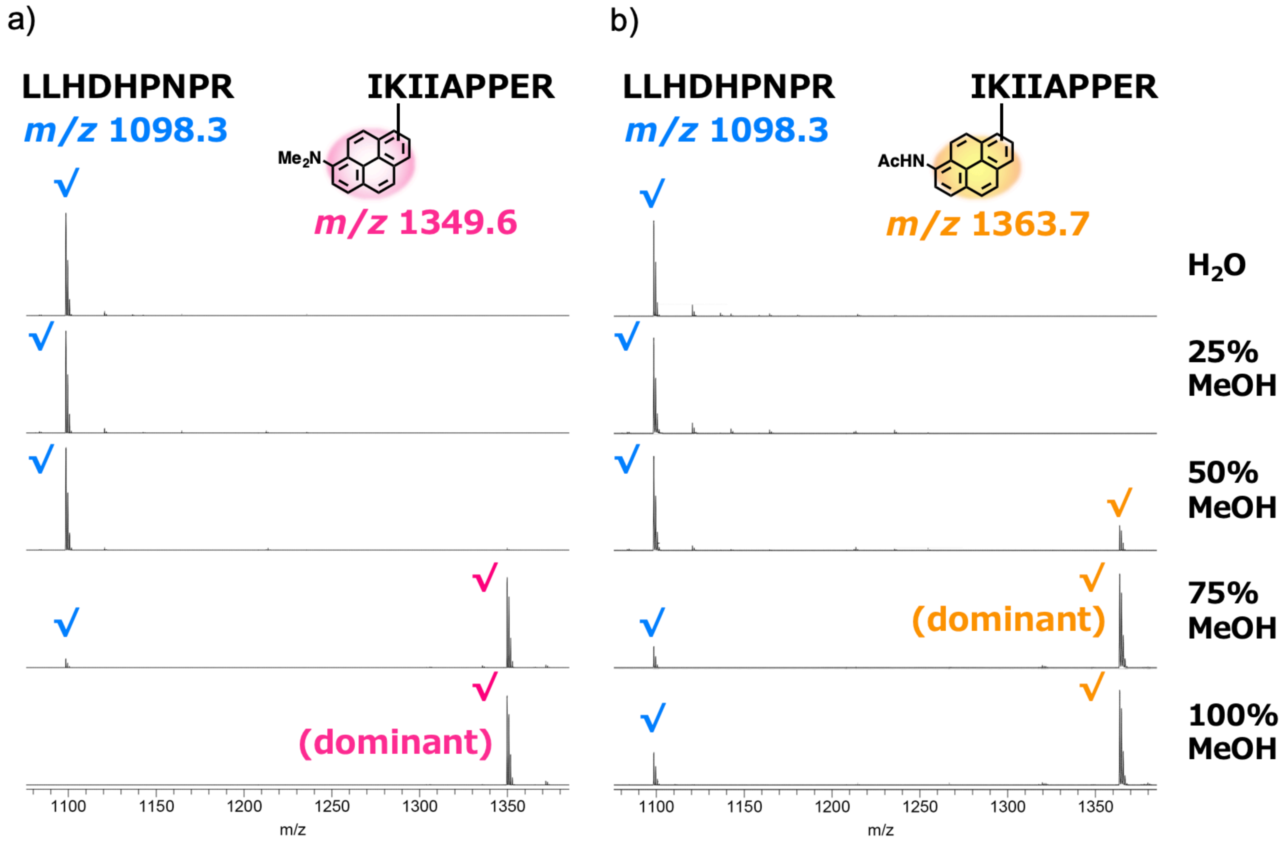

Figure S3. MALDI MS of the affinity-purified pyrene-labeled peptides using the TSK-G3000S gel. Samples were eluted stepwise by aq. MeOH (0–100%). (a) Mixture of a non-labeled peptide [LLHDHPNPR, *m/z* 1098.3 $(M+H)^+$] (400 pmol) and dmpy-labeled peptide [IK(dmpy)IIAPPER, *m/z* 1349.6 $(M+H)^+$] (10 pmol). (b) Mixture of a non-labeled peptide (400 pmol) and apy-labeled peptide [IK(apy)IIAPPER, *m/z* 1363.7 $(M+H)^+$] (30 pmol). dmpy- and apy-labeled peptides were mainly detected in the MeOH and 75% aq. MeOH fractions, respectively.

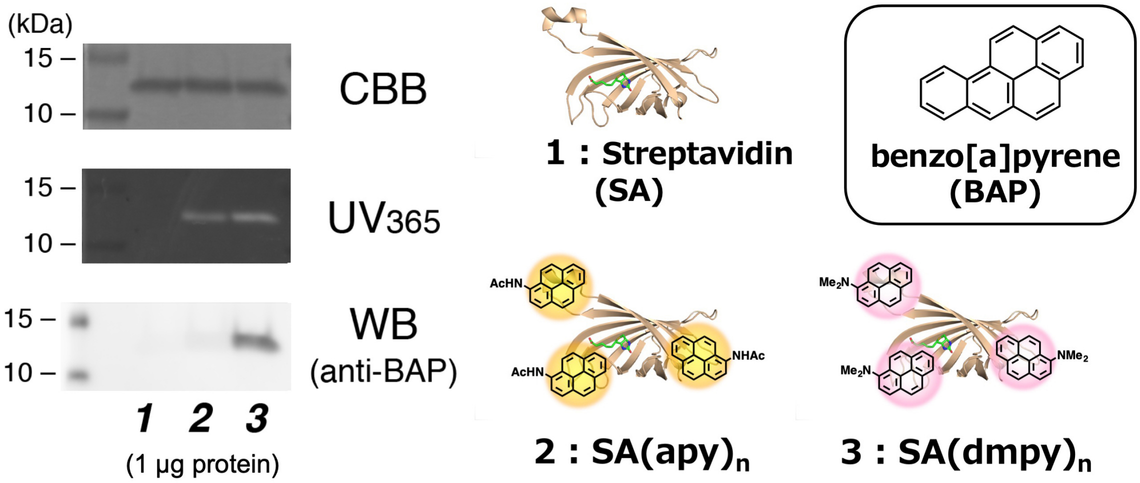

Figure S4. Selective detection of dmpy-labeled protein. Streptavidin (SA) and apy- or dmpy-labeled SA were detected with CBB (Coomassie brilliant blue) stain, fluorescence (UV365 nm), and immunoblotting analysis using anti-benzo[a]pyrene (anti-BAP) antibody.

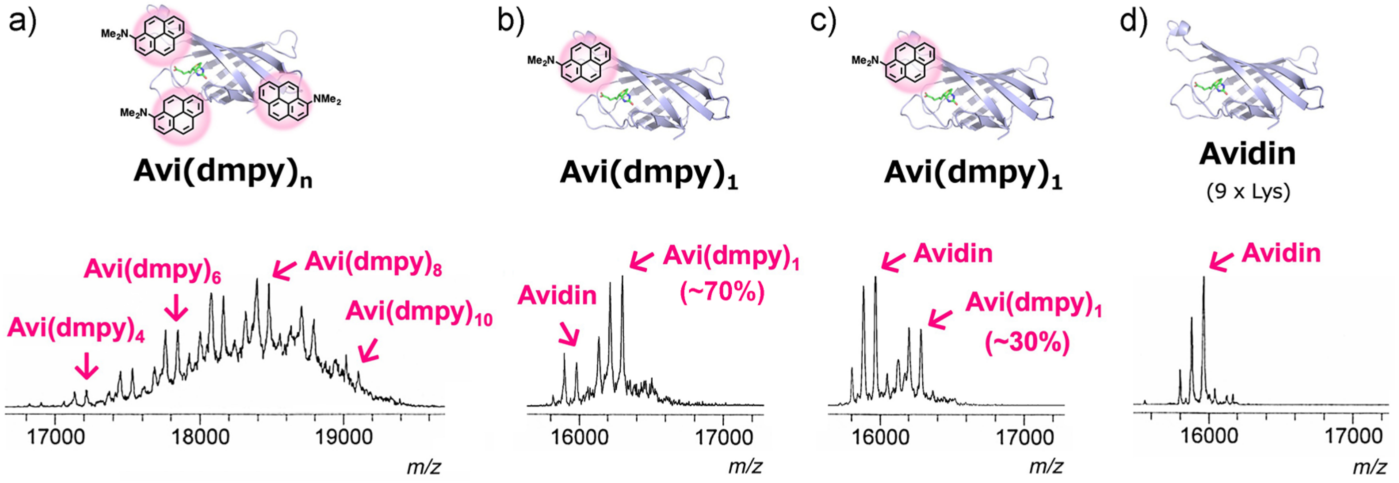

Figure S5. MALDI MS of avidin reacted with (a) dmpy–OSu (**4**), (b) dmpy–OSu–biotin (**5**), or (c) dmpy biotin probe **7**, and (d) unreacted avidin. Arrows indicate native avidin (15,960 Da, a major peak) and probe-labeled avidin (313 Da increase per dmpy molecule), respectively. Due to multiple *N*-glycosylation of native avidin, arrows were only shown for the representative ones for each mass spectrum.

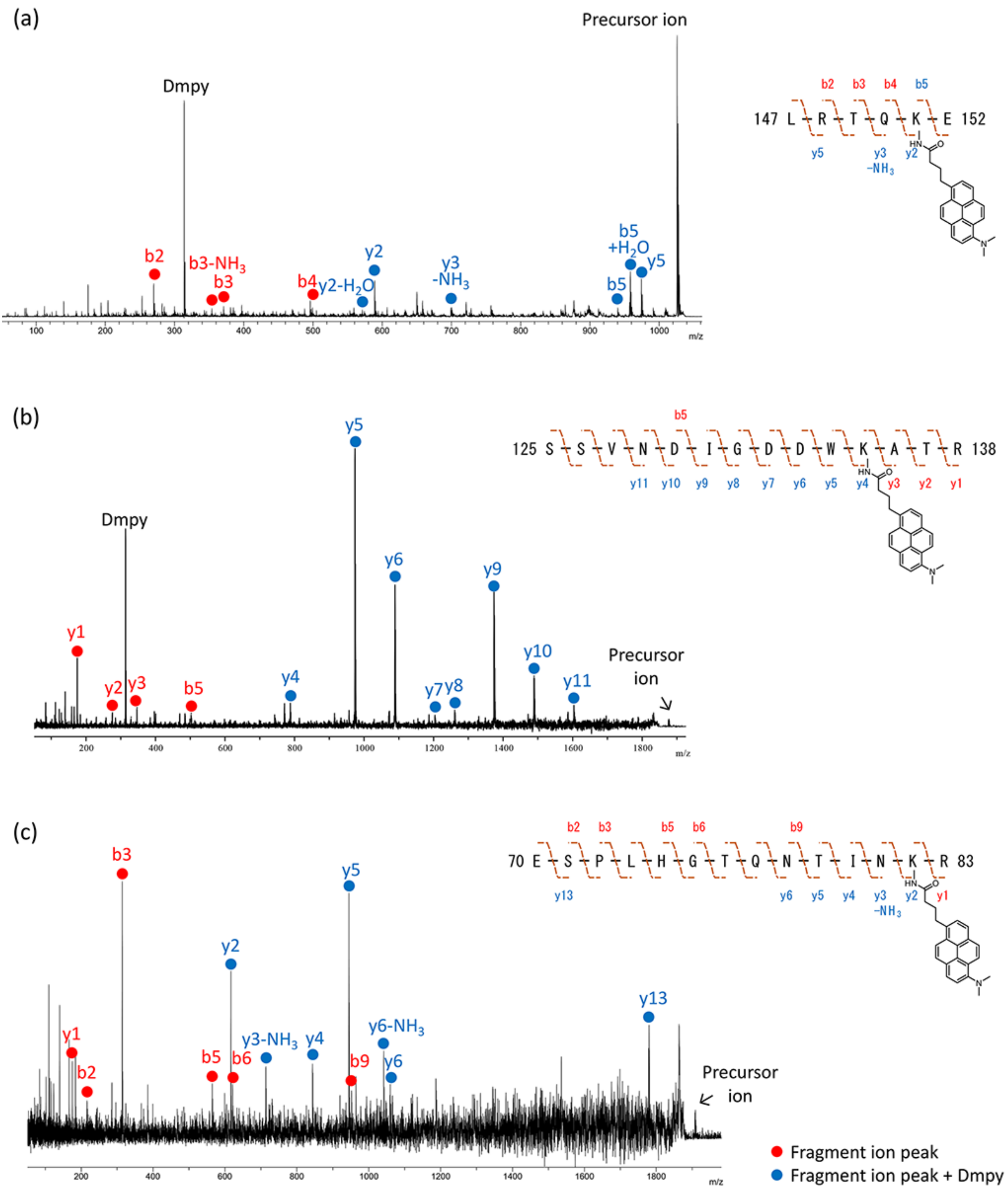

Figure S6. MALDI MS/MS analysis of the tryptic peptides of dmpy-labeled avidin. (a) K^{151}-labeled peptide [No. 1, precursor ion: *m/z* 1087.6 $(M+H)^+$]. (b) K^{135}-labeled peptide [No. 6, precursor ion: *m/z* 1876.9 $(M+H)^+$]. (c) K^{82}-labeled peptide [No. 7, precursor ion: *m/z* 1908.0 $(M+H)^+$].

Original gel images and blots on this manuscript (Cropped area are shown in red squares.)

Fig. 3b

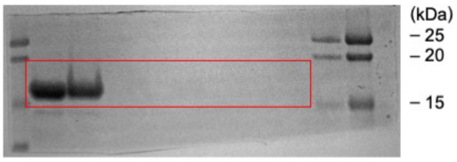

Fig. 3c

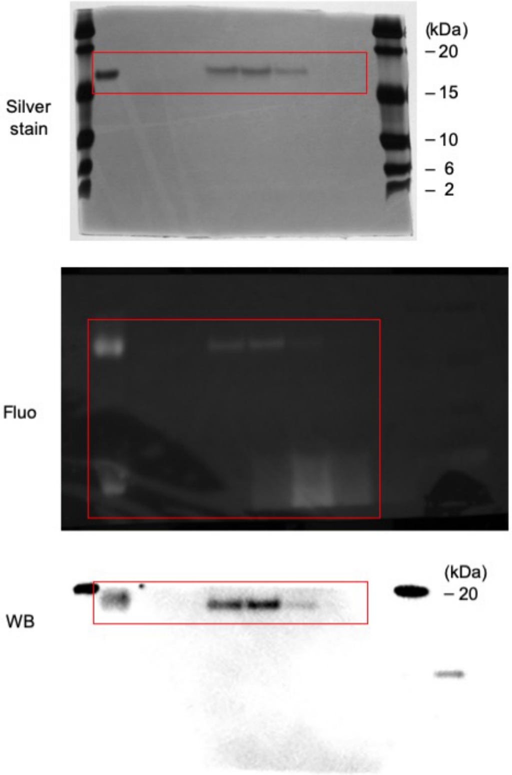

Fig. 5g

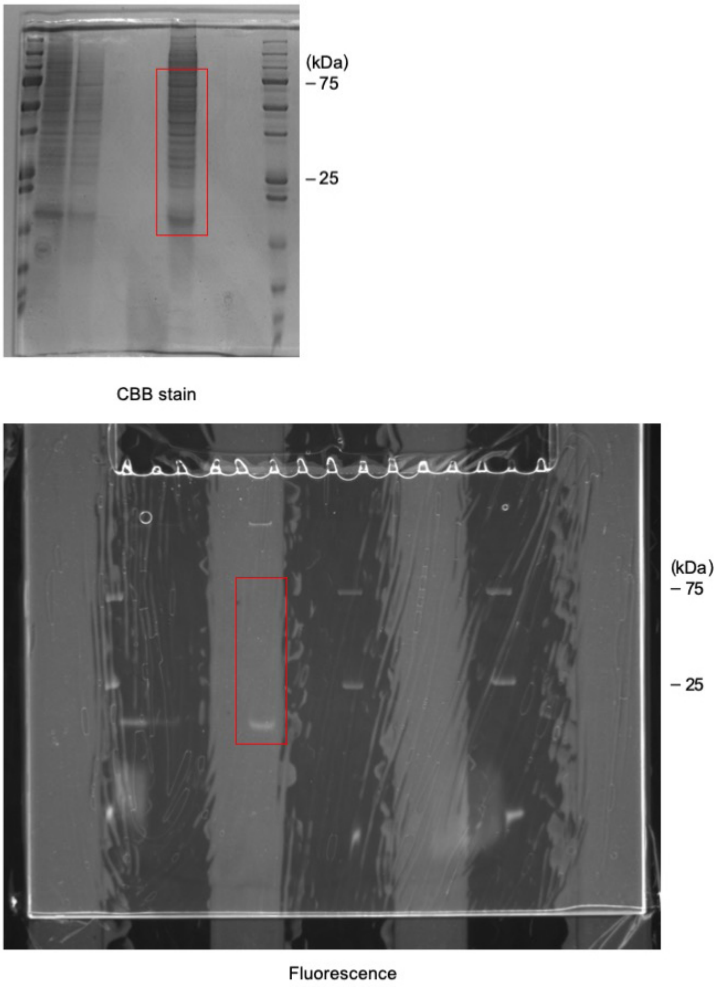

Fig. S4

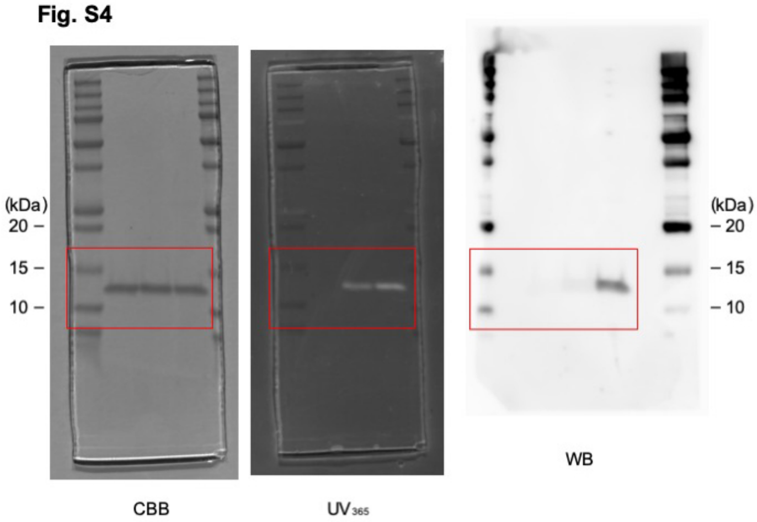

Materials and methods

General. NMR spectra were recorded on a Bruker Biospin AVANCE 400 spectrometer (400 MHz for ^{1}H and 100 MHz for ^{13}C). Chemical shifts are reported in parts per million (ppm) relative to the solvent peaks, δ_H 7.26 (residual C<u>H</u>Cl$_3$), δ_H 2.50 (residual C<u>H</u>D$_2$S(O)CD$_3$), δ_C 77.0 (<u>C</u>DCl$_3$), and δ_C 39.5 [(<u>C</u>D$_3$)$_2$S=O], respectively. Coupling constants (*J*) are shown in hertz. IR spectra were recorded on a JASCO FT/IR-230 spectrometer. High-resolution electrospray ionization mass spectra (HR-ESIMS) were measured on an Agilent 6120 TOF spectrometer or a JEOL AccuTOF CS spectrometer. UV-Vis spectra were recorded by a JASCO V-650 spectrometer. Solution and solid fluorescence spectra were recorded by a JASCO F8300 spectrofluorometer and a Hitachi F4500 spectrofluorometer, respectively. Quantum fluorescence yields were obtained by a Hamamatsu Photonics C9920-02 absolute PL quantum yield spectrometer. All chemicals were used as obtained commercially unless otherwise noted. Solid-phase peptide synthesis of *N*-α-Fmoc protected linear peptides was performed by Invitrogen Co., and synthetic products were purified by reversed-phase HPLC to give >99% purity grade samples. Organic solvents and reagents for moisture-sensitive reactions were distilled by the standard procedure. Fuji Silysia silica gels BW-820MH and FL60D were used for column chromatography. Merck precoated silica gel 60 F254 plates were used for thin layer chromatography (TLC).

Synthesis and spectroscopic data of dmpy probes

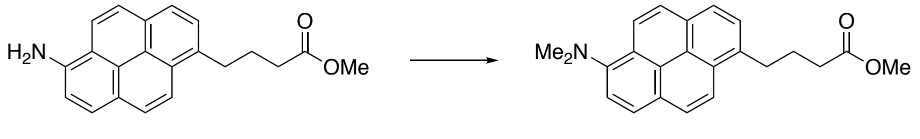

9 **1**

***N,N*-dimethylaminopyrene 1.** To a stirred solution of 6-aminopyrene **9** (345 mg, 1.09 mmol) [22] in acetonitrile (12 mL) were added formaldehyde 37 wt. % in water (0.45 mL, 5.5 mmol) and sodium cyanoborohydride (123 mg, 1.96 mmol). After stirring for 15 min at room temperature, acetic acid (0.2 mL) was added, and the resulting mixture was further stirred for 85 min. CHCl$_3$ (50 mL) was added, and the resulting mixture was washed with sat. NaHCO$_3$ aq. and brine, dried with Na$_2$SO$_4$, and concentrated. The crude material was purified with a SiO$_2$ column (70 g, hexane / CHCl$_3$ = 1/4) to give *N,N*-dimethylaminopyrene **1** (321 mg, 86%) as a brown oil. **1**: R_f = 0.50 (CHCl$_3$); UV (MeOH) λ_{max} 203 (ε 30000), 242 (ε 39000), 282 (ε 24000), 357 (ε 19000) nm; IR (CHCl$_3$) 3734, 3019, 2977, 1733, 1653, 1540, 1507, 1214, 929 cm^{-1}; ^{1}H NMR (400 MHz, CDCl$_3$) δ 8.42 (d, *J* = 8.7 Hz, 1H), 8.14 (d, *J* = 8.2 Hz, 1H), 8.09 (d, *J* = 8.2 Hz, 1H), 8.06 (d, *J* = 8.0 Hz, 1H), 8.04 (d, *J* = 8.2 Hz, 1H), 8.02 (d, *J* = 8.7 Hz, 1H), 7.82 (d, *J* = 7.8 Hz, 1H), 7.75 (d, *J* = 8.2 Hz, 1H), 3.70 (s, 3H), 3.36 (t, *J* = 7.7 Hz, 2H), 3.05 (s, 6H), 2.47 (t, *J* = 7.7 Hz, 2H), 2.20 (tt, *J* = 7.7, 7.7 Hz, 2H); ^{13}C NMR (100 MHz, CDCl$_3$) δ 173.9, 148.8, 134.9, 130.1, 129.1, 127.4, 127.3, 126.8, 126.4, 126.3, 125.6, 125.0, 124.7, 124.1, 122.7, 121.4, 116.4, 51.5, 45.6 (2C), 33.6, 32.8, 26.6; HRMS (ESI) *m/z* 346.1796 (calcd for $C_{23}H_{24}NO_2$ [M+H]$^+$, Δ –0.6 mmu).

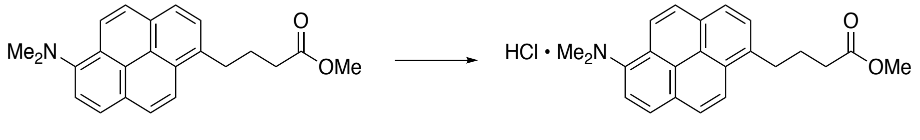

1 **1·HCl**

***N,N*-dimethylaminopyrene hydrochloride 1·HCl.** A solution of *N,N*-dimethylaminopyrene **1** (13.3 mg, 38.5 μmol) in 4 M hydrogen chloride solution in 1,4-dioxane (0.4 mL) was stirred for 30 min at room temperature. The reaction mixture was azeotropically concentrated with toluene to give *N,N*-dimethylaminopyrene hydrochloride **1·HCl** (14.7 mg, quant.) as a light

brown amorphous solid. **1·HCl**: R_f = 0.05 ($CHCl_3$ / acetone = 9/1); mp 152.5–153.0 °C; UV (MeOH) λ_{max} 204 (ε 24000), 242 (ε 39000), 282 (ε 22000), 357 (ε 19000) nm; IR ($CHCl_3$) 3733, 3019, 2976, 1733, 1653, 1540, 1507, 1215, 929 cm^{-1}; ^1H NMR (400 MHz, $CDCl_3$) δ 14.08 (br s, 1H), 9.19 (br s, 1H), 8.42 (d, *J* = 9.3 Hz, 1H), 8.33 (d, *J* = 9.3 Hz, 1H), 8.21 (d, *J* = 7.8 Hz, 1H), 8.21 (d, *J* = 7.5 Hz, 1H), 8.14 (br s, 1H), 8.09 (d, *J* = 9.3 Hz, 1H), 7.95 (d, *J* = 7.8 Hz, 1H), 3.69 (s, 3H), 3.55 (s, 6H), 3.40 (t, *J* = 7.8 Hz, 2H), 2.47 (t, *J* = 7.2 Hz, 2H), 2.17 (tt, *J* = 7.2, 7.8 Hz, 2H); ^{13}C NMR (100 MHz, $CDCl_3$) δ 173.7, 138.0, 134.8, 132.0, 130.9, 129.1, 128.7, 128.6, 126.6, 126.5, 125.7, 125.3, 124.8, 124.4, 123.8, 119.6, 116.7, 51.6, 47.6 (2C), 33.5, 32.9, 26.8.

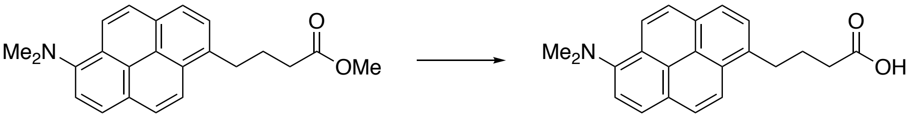

1 **10**

Carboxylic acid 10. To the solution of *N,N*-dimethylaminopyrene **1** (19.3 mg, 55.9 μmol) in THF (2 mL) was added 1 M LiOH aq. (2 mL). After being stirred for 24 h at room temperature, the reaction mixture was acidified with 1 M HCl aq. and extracted with $CHCl_3$ (5 mL × 4). The combined extracts were washed with brine, dried with Na_2SO_4, and concentrated to give carboxylic acid **10** (18.5 mg, quant.) as a brown oil. **10**: R_f = 0.19 ($CHCl_3$ / acetone = 9/1); IR ($CHCl_3$) 3160 (br), 3023, 2989, 1710, 1603, 1499, 1302 cm^{-1}; ^1H NMR (400 MHz, DMSO-d_6) δ 8.32 (d, *J* = 9.2 Hz, 1H), 8.20 (d, *J* = 9.2 Hz, 1H), 8.19 (d, *J* = 8.3 Hz, 1H), 8.14 (d, *J* = 7.8 Hz, 1H), 8.09 (d, *J* = 9.2 Hz, 2H), 7.88 (d, *J* = 7.8 Hz, 1H), 7.81 (d, *J* = 8.3 Hz, 1H), 3.35 (br s, 1H), 3.29 (t, *J* = 7.5 Hz, 2H), 2.98 (s, 6H), 2.37 (t, *J* = 7.2 Hz, 2H), 1.99 (tt, *J* = 7.2 Hz, 2H); ^{13}H NMR (100 MHz, DMSO-d_6) δ 174.4, 148.7, 135.5, 129.5, 128.6, 127.6, 127.3, 126.3, 126.1, 125.5, 125.3, 124.9, 124.2, 123.7, 122.4, 121.3, 116.8, 45.2 (2C), 33.4, 32.1, 26.7; HRMS (ESI) *m/z* 332.1642 (calcd for $C_{22}H_{22}NO_2$ $[M+H]^+$, Δ –0.3 mmu).

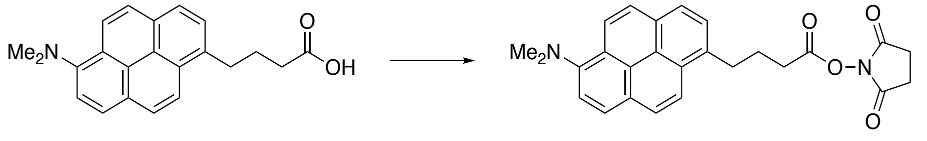

10 **4**

Dmpy–OSu (4). A solution of *N*-hydroxysuccinimide (NHS) (2.6 mg, 23 μmol) and EDC·HCl (4.3 mg, 23 μmol) in dry DMF (0.3 mL) was added to carboxylic acid **10** (5.0 mg, 15 μmol). After being stirred for 20 h at room temperature, the resulting mixture was azeotropically concentrated with toluene *in vacuo*, suspended in $CHCl_3$ (4 mL), and washed with brine, dried with Na_2SO_4, and concentrated. The crude material was purified with a SiO_2 column (0.5 g, $CHCl_3$ / acetone = 1/0 to 10/1) to give dmpy–OSu (**4**) (5.3 mg, 82%) as a light yellow oil. **4**: R_f = 0.63 ($CHCl_3$ / MeOH = 10/1); IR ($CHCl_3$) 3030, 2944, 2868, 2832, 2790, 1815, 1788, 1741, 1603, 1595, 1240, 1046 cm^{-1}; ^1H NMR (400 MHz, $CDCl_3$) δ 8.41 (d, *J* = 9.2 Hz, 1H), 8.13 (d, *J* = 9.2 Hz, 1H), 8.10 (d, *J* = 8.3 Hz, 1H), 8.06 (d, *J* = 7.8 Hz, 1H), 8.03 (d, *J* = 9.1 Hz, 1H), 8.02 (d, *J* = 9.2 Hz, 1H), 7.84 (d, *J* = 7.8 Hz, 1H), 7.74 (d, *J* = 8.3 Hz, 1H), 3.44 (t, *J* = 7.6 Hz, 2H), 3.04 (s, 4H), 2.86 (br s, 6H), 2.73 (t, *J* = 7.2 Hz, 2H), 2.30 (tt, *J* = 7.2, 7.6 Hz, 2H); ^{13}C NMR (100 MHz, $CDCl_3$) δ 169.1 (2C), 168.6, 140.2, 134.1, 130.3, 129.1, 127.6, 127.6, 126.8, 126.4, 126.3, 125.6, 125.1, 124.6, 124.2, 122.8, 121.3, 116.5, 45.7 (2C), 32.3, 30.5, 26.3, 25.6 (2C); HRMS (ESI) *m/z* 429.1810 (calcd for $C_{26}H_{25}N_2O_4$ $[M+H]^+$, Δ +0.1 mmu).

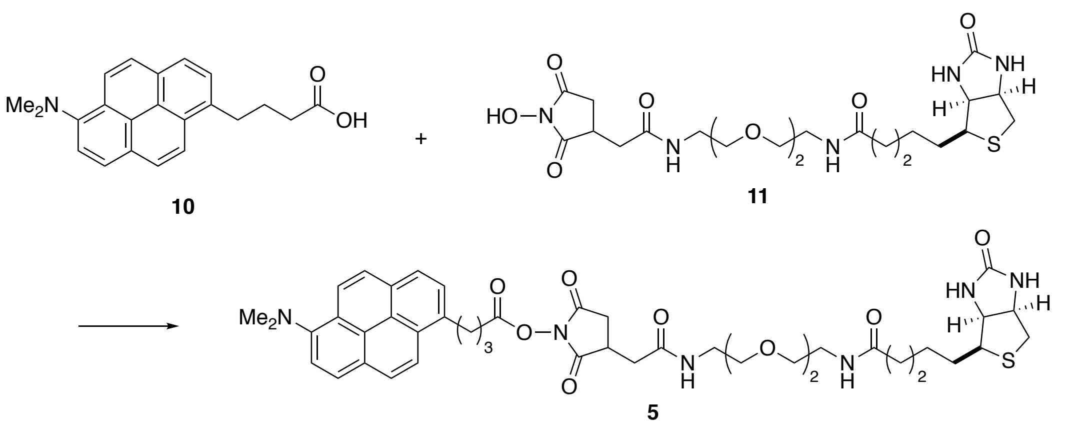

dmpy–OSu–biotin (5). To a stirred solution of carboxylic acid **10** (6.7 mg, 20 μmol) and HOSu–biotin (**11**) [25] (9.8 mg, 19 μmol) in dry DMF (0.3 mL) were added EDC·HCl (8.9 mg, 46 μmol) and HOBt (7.5 mg, 56 μmol) under a nitrogen atmosphere. After being stirred for 48 h at room temperature, the resulting mixture was azeotropically concentrated with toluene, diluted with $CHCl_3$ (7 mL), washed with brine, dried and concentrated. The crude material was purified with a SiO_2 column chromatography (FL60D 0.5 g, $CHCl_3$/acetone = 1/0 to 1:1, and MeOH) to give dmpy–OSu–biotin (**5**) 12 mg (77%) as a lightyellow oil. **5**: R_f = 0.55 ($CHCl_3$ / MeOH = 4/1); IR ($CHCl_3$) 3443, 3317, 3033, 3006, 2941, 2870, 1739, 1702, 1662, 1524, 1458, 1235, 1055, 849 cm^{-1}; ^{1}H NMR (400 MHz, DMSO-d_6) δ 8.33 (d, J = 9.1 Hz, 1H), 8.33 (s, 1H), 8.22 (d, J = 9.2 Hz, 1H), 8.20 (d, J = 8.0 Hz, 1H), 8.15 (d, J = 8.3 Hz, 1H), 8.15 (m, 1H), 8.10 (d, J = 9.2 Hz, 1H), 7.91 (d, J = 8.0 Hz, 1H), 7.85 (m, 1H), 7.81 (d, J = 8.3 Hz, 1H), 6.42 (s, 1H), 6.36 (s, 1H), 4.28 (dd, J = 7.6, 5.1 Hz, 1H), 4.09 (m, 1H), 3.42–3.35 (m, 8H), 3.24–3.13 (m, 4H), 3.09–2.98 (m, 3H), 2.98 (s, 6H), 2.85 (t, J = 7.0 Hz, 2H), 2.79 (dd, J = 12.5, 5.0 Hz, 1H), 2.71–2.60 (m, 2H), 2.56 (br d, J = 12.5 Hz, 1H), 2.10–2.01 (m, 4H), 1.59 (m, 1H), 1.53–1.39 (m, 5H), 1.33–1.21 (m, 3H); ^{13}C NMR (100 MHz, $CDCl_3$) δ 173.4, 172.1, 169.5, 169.0, 168.7, 163.8, 149.0, 134.1, 130.3, 129.1, 128.3, 127.6, 126.7, 126.4, 126.2, 125.6, 125.2, 124.6, 124.2, 122.9, 121.2, 116.5, 77.2, 70.1, 69.9, 68.0 (2C), 61.6, 60.1, 55.2, 45.7 (2C), 40.9, 40.4, 39.3, 39.1, 35.8, 32.3, 30.6, 27.8, 26.4, 25.6 (2C), 25.3; HRMS (ESI) m/z 865.3576 (calcd for $C_{44}H_{54}N_6NaO_9S$ $[M+Na]^+$, Δ +1.2 mmu).

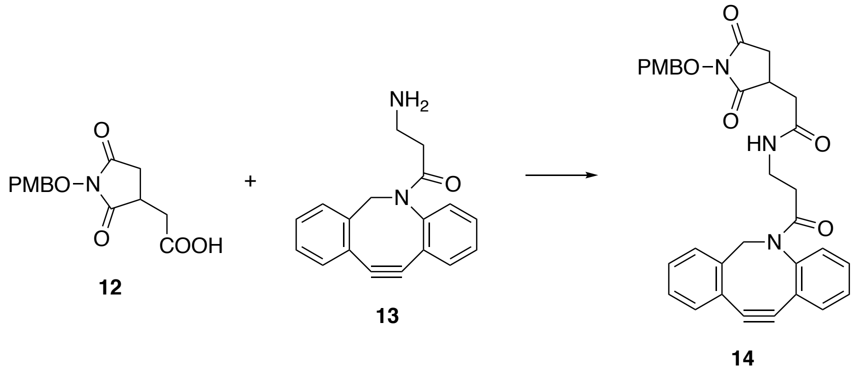

Amide 14. To a solution of carboxylic acid **12** (6.9 mg, 23 μmol) [25] in dry DMF (0.2 mL) were added HOBt (6.1 mg, 45 μmol), EDC·HCl (6.9 mg, 36 μmol), and dibenzocyclooctyne amine **13** (5.0 mg, 18 μmol).[S1] After being stirred for 20 h at room temperature, the reaction mixture was azeotropically concentrated with toluene *in vacuo*, suspended in $CHCl_3$ (4 mL), and washed with sat. NH_4Cl and brine, dried with Na_2SO_4, and concentrated. The crude material was purified with a SiO_2 column (0.5 g, $CHCl_3$ / acetone = 9/1, 4/1 to 2/1) to give amide **14** (9.9 mg, quant.) as a colorless oil. **14**: R_f = 0.66 ($CHCl_3$ / MeOH 9/1); IR ($CHCl_3$) 3437, 3007, 2935, 2840, 1787, 1725, 1667, 1613, 1516, 1441, 1401, 1252, 1176, 1064 cm^{-1}; ^{1}H NMR (400 MHz, $CDCl_3$) δ 7.67 (d, J = 7.3 Hz, 1H), 7.44–7.26 (m, 7H), 7.42 (d, J = 8.7 Hz, 2H), 6.88 (d, J = 8.8 Hz, 2H), 6.10 (br s, 1H), 5.13 (d, J = 7.0 Hz, 1H), 5.05 (s, 2H), 3.81 (s, 3H), 3.71 (d, J = 7.0 Hz, 1H), 3.34 (m, 1H), 3.18 (m, 1H), 2.92 (m, 1H), 2.78 (ddd, J = 18.0, 9.1, 4.6 Hz, 1H), 2.50 (br dd, J = 16.3, 4.2 Hz, 1H), 2.47–2.29 (m, 3H), 1.96 (m, 1H); ^{13}C NMR (100 MHz, $CDCl_3$) δ 173.6 [173.5], 172.1 [172.1], 170.7 [170.7], 168.7, 160.4 [160.4], 150.9 [150.9], 147.9 [147.9], 132.0

[132.1], 131.7 [131.6] (2C), 129.0 [129.0], 128.7 [128.7], 128.5 [128.4], 127.9, 127.3 [127.3], 125.7 [125.6], 122.8, 122.5 [122.4], 114.7 [114.8], 113.8 (2C), 107.8, 78.1, 77.2, 55.5, 55.3, 35.9, 35.5, 35.4 [35.4], 34.5 [34.5], 33.7 [33.7], 31.9 [31.9]. Signals derived from minor rotamer (ca. 10:9 ratio) were shown in parenthesis (square blankets); HRMS (ESI) *m/z* 574.1945 (calcd for $C_{32}H_{29}N_3NaO_6$ $[M+Na]^+$, Δ –0.4 mmu).

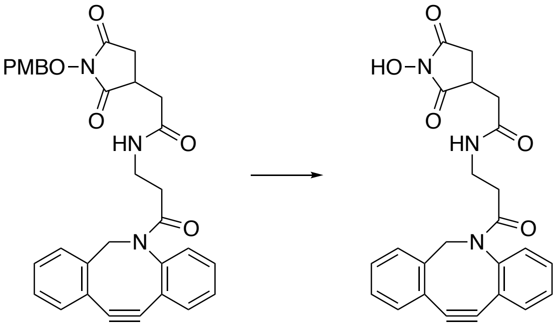

***N*-hydroxyimide 15.** A solution of amide **14** (4.5 mg, 8.2 µmol) in a 1:1 mixture of dry CH_2Cl_2 and trifluoroacetic acid (0.3 mL) was stirred for 10 min at room temperature, and then azeotropically concentrated with toluene *in vacuo*. The crude material was purified with a SiO_2 column (0.5 g, $CHCl_3$ / MeOH = 1/0, 49/1, 19/1 to 9/1) to give *N*-hydroxyimide **15** (2.9 mg, 82%) as a colorless oil. **15**: R_f 0.35 ($CHCl_3$ / MeOH = 9/1); IR ($CHCl_3$) 3432 (br), 3012, 2931, 2859, 2158, 1786, 1718, 1663, 1520, 1440 cm^{-1}; ^1H NMR (400 MHz, DMSO-d_6) δ 10.54 (s, 1H), 7.81 (m, 1H), 7.63 (d, *J* = 7.2 Hz, 1H), 7.61–7.52 (m, 2H), 7.52–7.45 (m, 2H), 7.39 (t, *J* = 7.5 Hz, 1H), 7.35 (t, *J* = 7.5 Hz, 1H), 7.31 (t, *J* = 7.4 Hz, 1H), 5.04 (d, *J* = 13.8 Hz, 1H), 3.63 (d, *J* = 13.8 Hz, 1H), 3.08 (m, 1H), 3.00–2.88 (m, 2H), 2.67 (ddd, *J* = 17.5, 9.0, 4.0 Hz, 1H), 2.47–2.31 (m, 3H), 2.24 (ddd, *J* = 17.5, 9.3, 4.5 Hz, 1H), 1.82 (m, 1H); ^{13}C NMR (100 MHz, DMSO-d_6) δ 174.8, 171.9, 170.1, 169.3, 148.4, 132.4, 129.5, 129.0, 128.2, 128.1, 127.7, 126.8, 125.2, 122.4, 121.5, 121.4, 81.4, 79.2, 54.9, 34.6, 34.1, 34.0, 33.2, 31.4; HRMS (ESI) *m/z* 454.1368 (calcd for $C_{24}H_{21}N_3NaO_5$ $[M+Na]^+$, Δ –0.5 mmu).

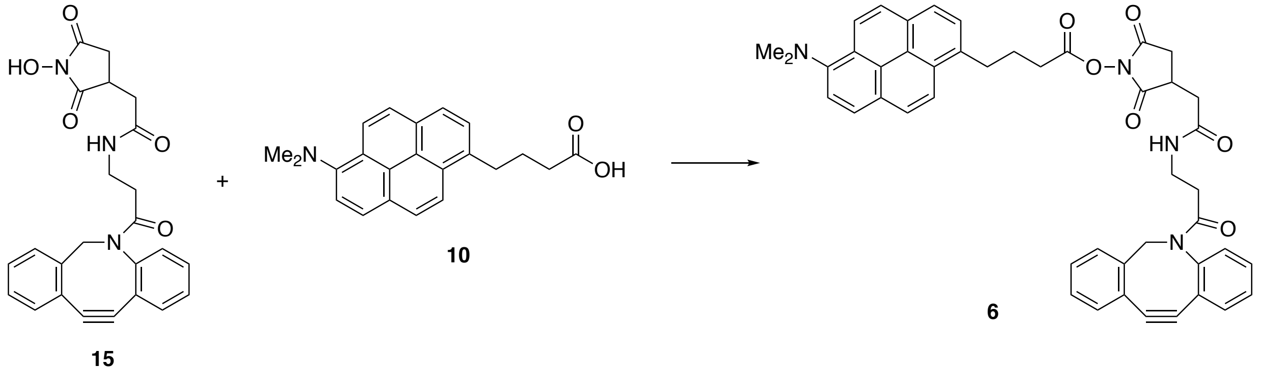

dmpy–OSu–DBCO (6). To a solution of carboxylic acid **10** (1.6 mg, 4.8 µmol) and *N*-hydroxyimide **15** (1.9 mg, 4.4 µmol) in dry DMF (0.15 mL) were added HOBt (2.0 mg, 15 µmol), and EDC·HCl (2.5 mg, 13 µmol). After being stirred for 35 h at room temperature, the reaction mixture was azeotropically concentrated with toluene *in vacuo*, suspended in $CHCl_3$ (4 mL), and washed with brine, dried with Na_2SO_4, and concentrated. The crude material was purified with a SiO_2 column (FL60D 0.5 g, $CHCl_3$ / MeOH = 1/0, 49/1, 19/1, 9/1 to 1/1) to give dmpy–OSu–DBCO (**6**) (2.0 mg, 61%) as a light yellow oil. **6**: R_f 0.18 ($CHCl_3$ / acetone = 9/1); IR ($CHCl_3$) 3437, 3010, 2942, 2867, 2833, 2790, 1786, 1739, 1671, 1516, 1300, 1232, 1068, 849 cm^{-1}; ^1H NMR (400 MHz, $CDCl_3$) δ 8.40 (d, *J* = 9.2 Hz, 1H), 8.13 (d, *J* = 9.2 Hz, 1H), 8.09 (d, *J* = 9.1 Hz, 1H), 8.06 (d, *J* = 8.4 Hz, 1H), 8.02 (m, 2H), 7.84 (d, *J* = 7.8 Hz, 1H), 7.74 (d, *J* = 8.4 Hz, 1H), 7.72–7.64 (m, 3H), 7.55–7.50 (m, 2H), 7.42–7.27 (m, 3H), 6.13 (br s, 1H), 5.12 (dd, *J* = 13.9, 5.7 Hz, 1H), 3.68 (dd, *J* = 13.9, 7.4 Hz, 1H), 3.44 (t, *J* = 7.7 Hz, 2H), 3.35 (m, 1H), 3.21 (m, 1H), 3.05 (s, 6H), 3.00 (m, 1H), 2.73 (t, *J* = 7.2 Hz, 2H), 2.55 (m, 1H), 2.44 (m, 1H), 2.35–2.26 (m, 3H),

1.99 (m, 1H), 1.68 (tt, J = 7.7, 7.2 Hz, 2H); ^{13}C NMR (100 MHz, $CDCl_3$) δ 171.7, 168.7 (2C), 168.6, 168.5, 148.9, 148.4, 134.2, 132.2, 130.3, 129.2, 129.0, 128.7, 128.5, 128.4, 128.4, 128.0, 127.6, 127.6, 127.3, 126.8, 126.4, 126.3, 125.7, 125.6, 125.1, 124.7, 124.2, 122.8, 122.4, 121.3, 116.5, 107.9, 79.3, 77.2, 55.5 [53.8], 45.7 (2C), 35.5, 34.6, 32.4, 31.7, 30.6, 29.7, 29.3, 26.4. Signals derived from minor rotamer were shown in parenthesis (square blankets); HRMS (ESI) *m/z* 767.2827 (calcd for $C_{46}H_{40}N_4NaO_6$ $[M+Na]^+$, Δ –1.3 mmu).

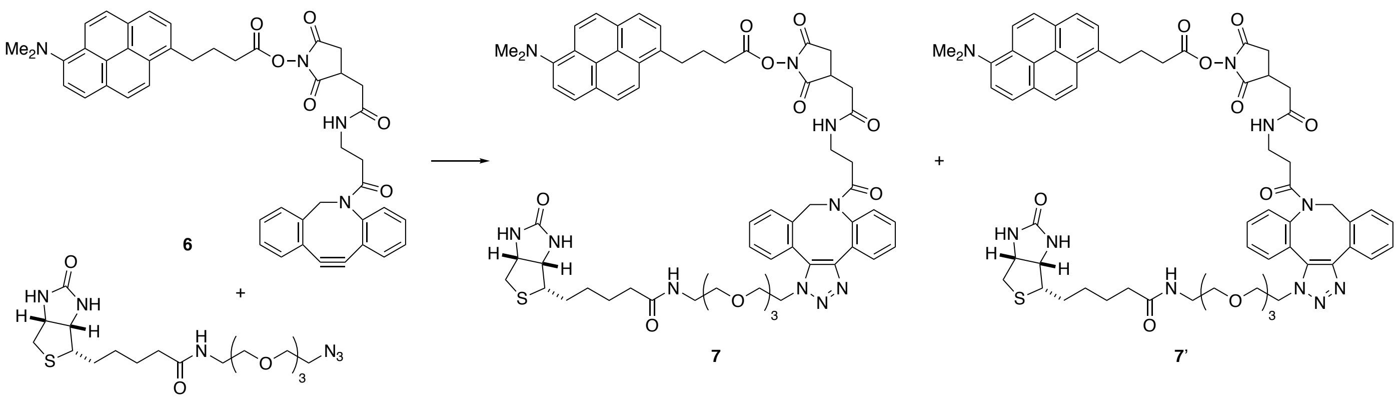

dmpy biotin probe 7. Solutions of 10 mM dmpy–OSu–DBCO (**6**) in DMSO (10 μL, 100 nmol) and 10 mM azide–PEG_3–biotin conjugate [Cat. No. 762024, Sigma–Aldrich] in DMSO (10 μL, 100 nmol) were mixed and stood for 12 h at room temperature to give a 5 mM solution of dmpy biotin probe **7** (quant., monitored by TLC analysis) as ca. 1:1 mixture of regioisomers with **7'**. **7**: R_f 0.38 ($CHCl_3$ / MeOH = 9/1); IR ($CHCl_3$) 3464, 3339 (br), 3004, 2935, 2870, 1738, 1704, 1660, 1209, 1054 cm^{-1}; ^{1}H NMR (400 MHz, DMSO-d_6) δ 8.33 (d, J = 8.8 Hz, 1H), 8.31 (s, 2H), 8.22 (d, J = 9.2 Hz, 1H), 8.20 (d, J = 8.2 Hz, 1H), 8.15 (d, J = 7.8 Hz, 1H), 8.10 (d, J = 9.2 Hz, 1H), 8.10 (d, J = 8.8 Hz, 1H), 7.92 (m, 2H), 7.83 (m, 2H), 7.81 (d, J = 8.2 Hz, 1H), 7.64 (m, 1H), 7.58–7.45 (m, 3H), 7.44–7.22 (m, 3H), 6.42 (br s, 1H), 6.35 (br s, 1H), 5.87 (m, 1H), 4.59 (m, 1H), 4.52–4.41 (m, 2H), 4.29 (m, 1H), 4.11 (m, 1H), 3.76 (m, 1H), 3.60 (t, J = 5.0 Hz, 2H), 3.56–3.47 (m, 8H), 3.30–3.28 (m, 2H), 3.17 (m, 2H), 3.14–3.01 (m, 2H), 2.98 (s, 6H), 2.90–2.74 (m, 2H), 2.63–2.51 (m, 2H), 2.15–1.98 (m, 6H), 1.89 (m, 1H), 1.66–1.40 (m, 6H), 1.35–1.21 (m, 4H); ^{13}C NMR (100 MHz, DMSO-d_6) δ 178.5, 174.3, 172.6, 172.2, 169.7 (2C), 162.7, 149.0, 148.8, 135.5, 134.9, 134.9, 131.6, 131.6, 130.0, 129.7, 129.5, 129.3, 129.3, 129.1, 128.9, 128.6, 128.3, 127.7, 127.5, 126.3, 126.1, 125.5, 125.4, 124.9, 124.3, 123.7, 122.6, 121.2, 116.9, 79.2, 70.6, 69.8, 69.8, 69.7, 69.6, 69.5, 69.3, 69.2, 69.1, 61.1, 59.2, 55.4, 50.0, 55.8, 45.3 (2C), 39.2, 39.0, 38.5, 35.1, 32.1, 31.6, 30.2, 29.6, 28.2, 28.1, 26.7, 25.3; HRMS (ESI) *m/z* 1211.5005 (calcd for $C_{64}H_{72}N_{10}NaO_{11}S$ $[M+Na]^+$, Δ +1.0 mmu).

Preparation of dmpy- and apy-labeled peptides. Solid-phase peptide synthesis of *N*-α-Fmoc protected or non-protected peptides was performed by Invitrogen Co., and synthetic products were purified by reversed-phase HPLC to give >99% purity grade samples. *N*-α-Fmoc protected linear peptide (360 nmol) was dissolved in 10 mM dmpy–OSu (**4**) or apy–OSu in DMSO (66 μL, 660 nmol) and 1 M *N*-methylmorpholine in DMSO (6.6 μL, 6.6 μmol). After standing at room temperature for 42 h, methylamine in 40% aqueous solution (5 μL, 60 μmol) was added, and the resulting mixture was stand for 8 h. To remove the Fmoc group, piperidine (10 μL) was added, and stand for 13 h. The reaction mixture was concentrated, suspended in 30% aq. MeCN / 0.1% TFA (200 μL), and filtered. The filtrate was concentrated and purified with a reversed-phase HPLC [Develosil ODS-HG-5 (ϕ 10 × 250 mm), 30% MeCN / 0.1% TFA, 2 mL/min, 280 nm] to give dmpy- and apy-labeled peptides as colorless oils. Samples were quantified by the analytical HPLC based on the UV (280 nm) and fluorescence ($\lambda_{ex/em}$ 365/407 nm). Preparation and characterization of a nonapeptide [LLHDHPNPR] (Fig. 3a) was conducted as described previously.[S2]

(a) **ANAWK(dmpy)STLVGHD** (Figure 2): yield, 36 nmol (10%); MS (MALDI) *m/z* 1611.8 $(M+H)^+$; HRMS (ESI) *m/z* 806.4019 (calcd for $C_{79}H_{108}N_{18}O_{19}$ $[M+2H]^{2+}$, Δ +0.2 mmu).

(b) **IK(dmpy)IIAPPER** (Figure 3a): yield, 90 nmol (25%); MS (MALDI) *m/z* 1349.9 $(M+H)^+$; HRMS (ESI) *m/z* 675.4017 (calcd for $(C_{70}H_{106}N_{14}O_{13})/2$ $[M+2H]^{2+}$, Δ –1.0 mmu).

(c) **IK(apy)IIAPPER** (Figure 3b): yield, 32 nmol (9%); MS (MALDI) *m/z* 1363.7 $(M+H)^+$; HRMS (ESI) *m/z* 682.3940 (calcd for $(C_{70}H_{104}N_{14}O_{14})$ $[M+2H]^{2+}$, Δ +1.7 mmu).

(d) **K(dmpy)ILTER** (Figure S2a): yield, 64 nmol (18%); MS (MALDI) *m/z* 1072.7 $(M+H)^+$; HRMS (ESI) *m/z* 536.8139 (calcd for $(C_{55}H_{83}N_{11}O_{11})$ $[M+2H]^{2+}$, Δ +0.7 mmu).

(e) **K(apy)ILTER** (Figure S2b): yield, 120 nmol (33%); MS (MALDI) *m/z* 1086.8 $(M+H)^+$; HRMS (ESI) *m/z* 543.8008 (calcd for $(C_{55}H_{81}N_{11}O_{12})$ $[M+2H]^{2+}$, Δ –2.0 mmu).

Supporting References

S1. Kuzmin, A., Poloukhtine, A., Wolfert, M. A. & Popik, V. V. Surface functionalization using catalyst-free azide-alkyne cycloaddition. *Bioconjugate Chem.* **21**, 2076–2085 (2010).

S2. Kita, M., Black, D. StC., Ohno, O., Yamada, K., Kigoshi, H. & Uemura, D. Duck-billed platypus venom peptides induce Ca^{2+} influx in neuroblastoma cells. *J. Am. Chem. Soc.* **131**, 18038–18039 (2009).

NMR spectra of new compounds

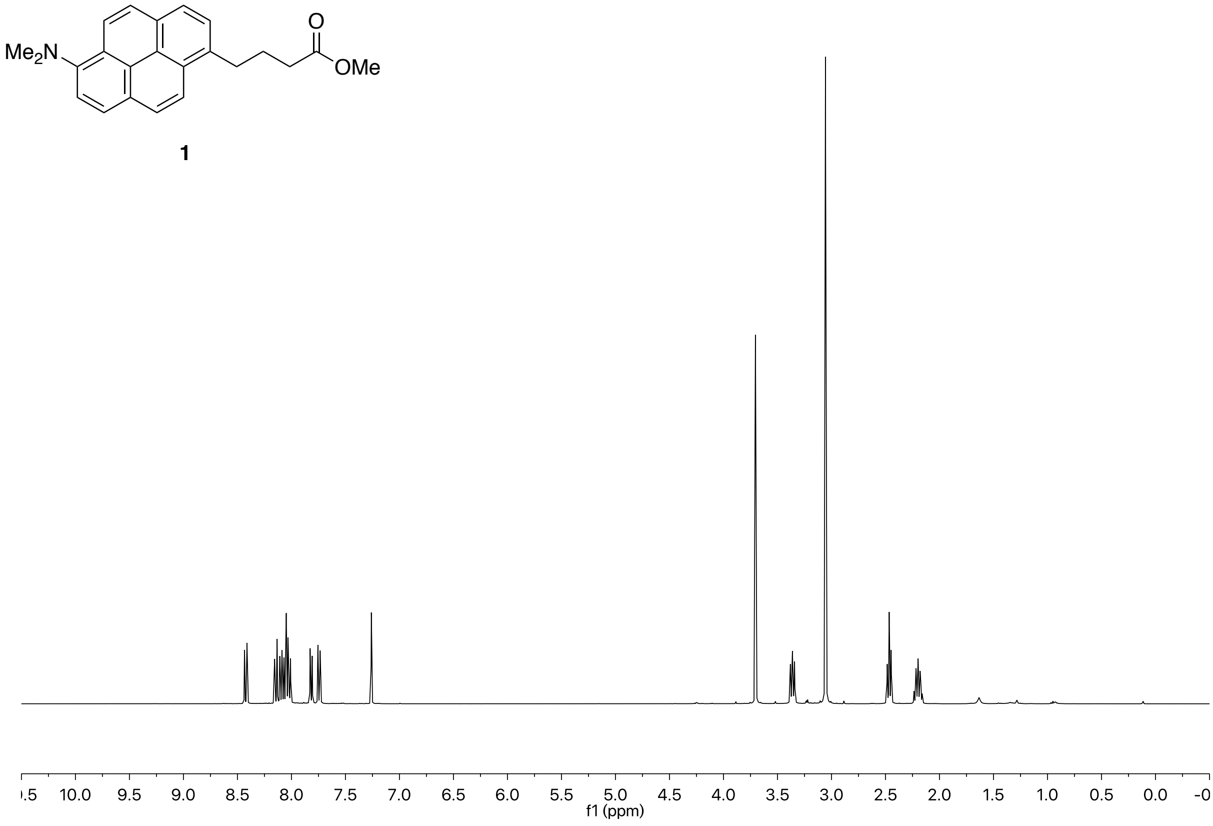

^{1}H NMR spectrum of **1** (400 MHz, $CDCl_3$).

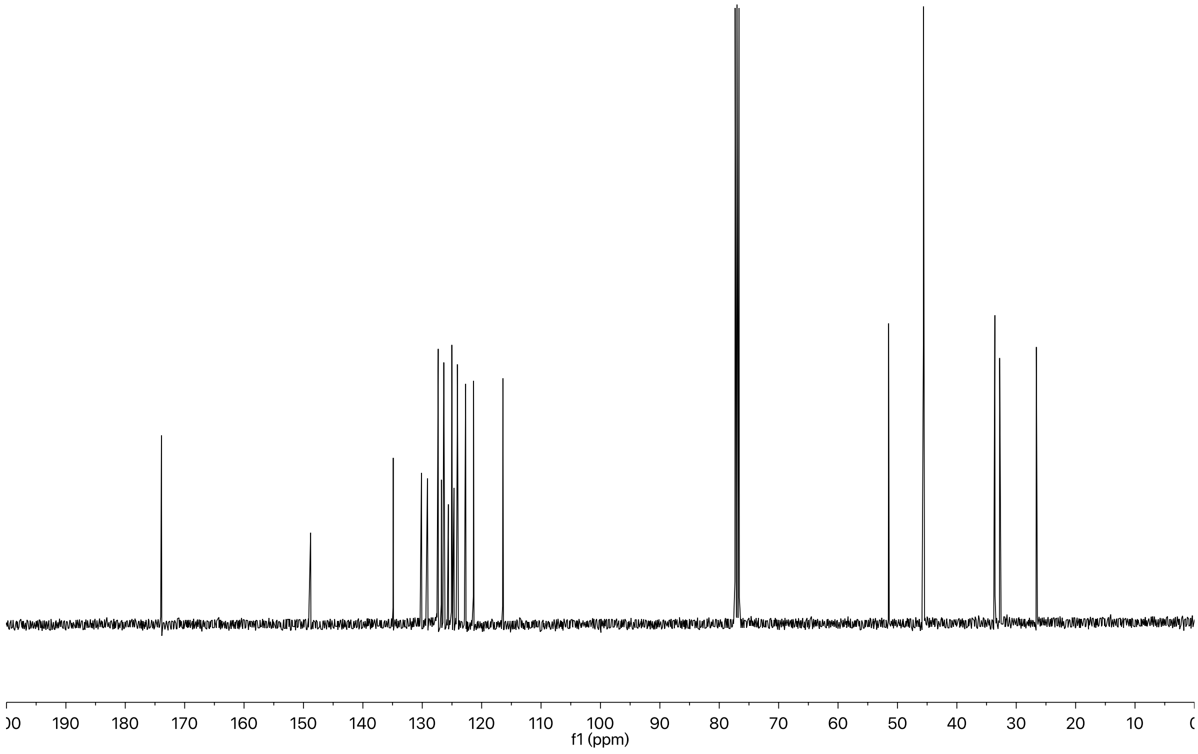

^{13}C NMR spectrum of **1** (100 MHz, $CDCl_3$).

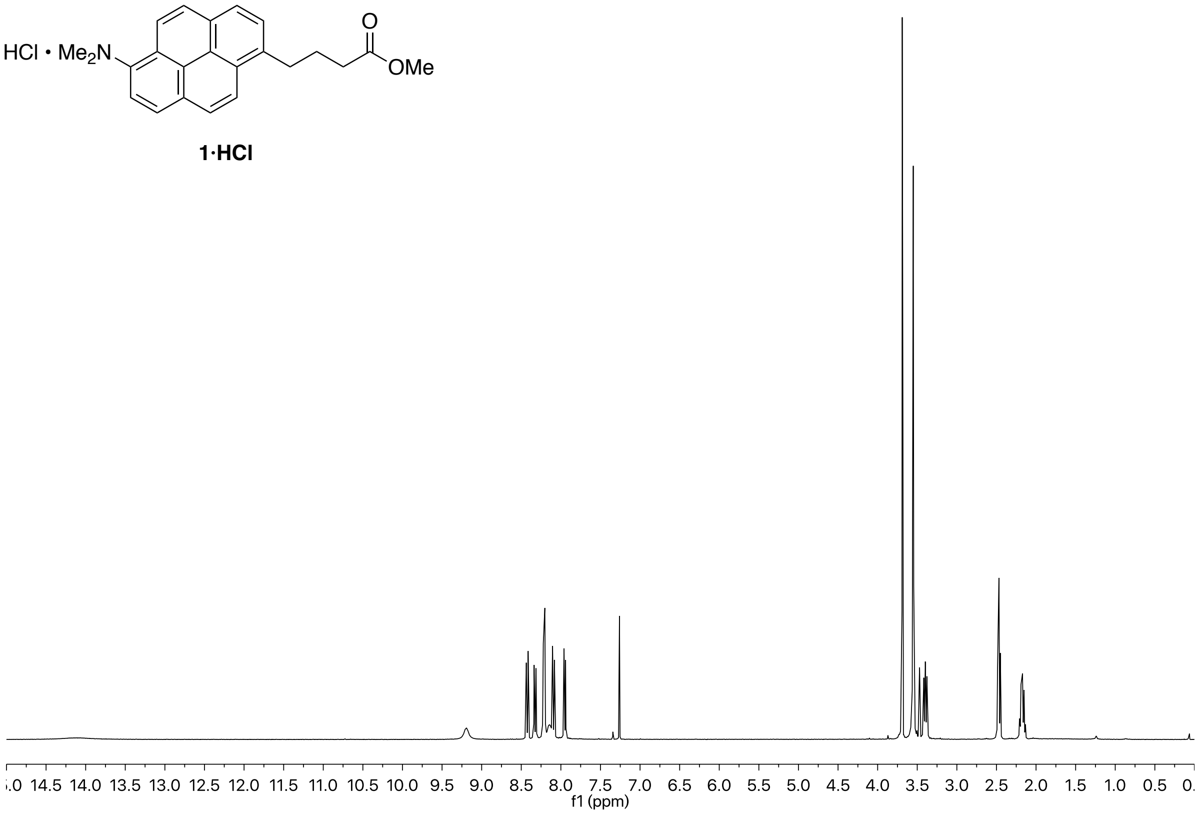

^{1}H NMR spectrum of **1·HCl** (400 MHz, $CDCl_3$).

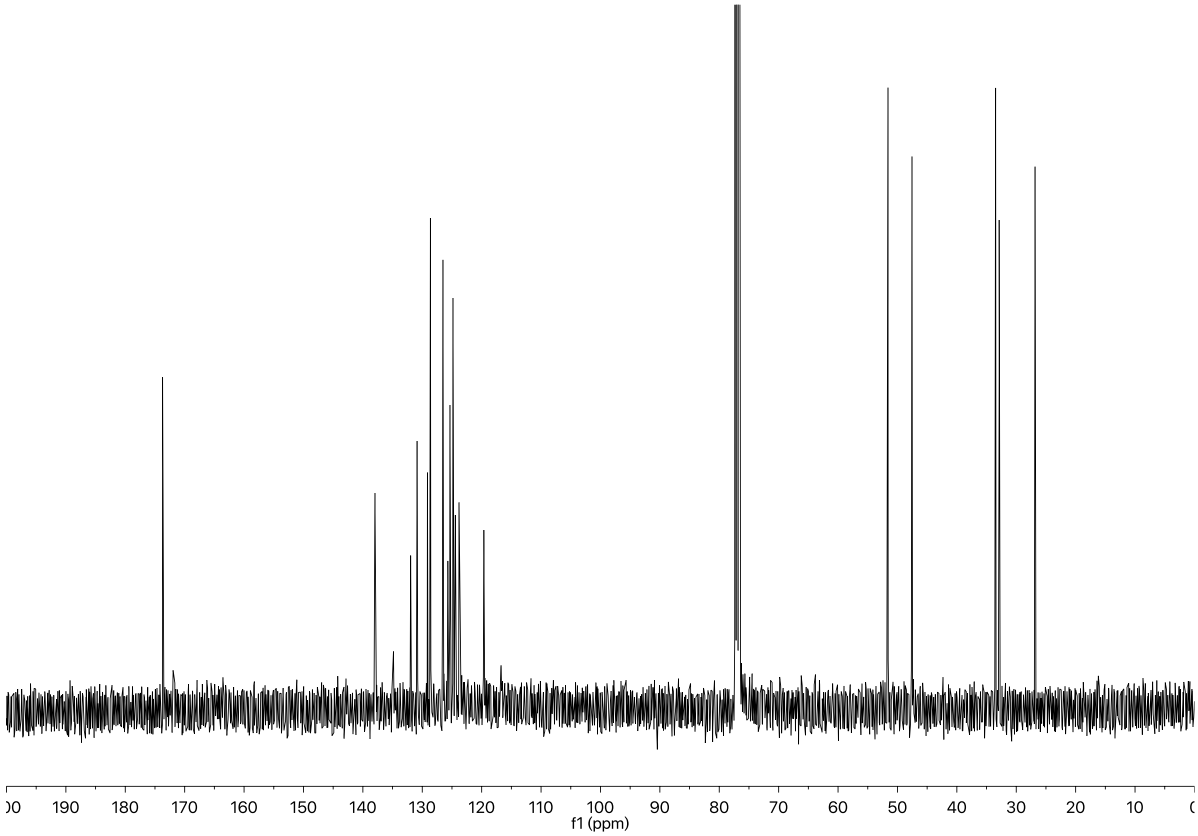

^{13}C NMR spectrum of **1·HCl** (100 MHz, $CDCl_3$).

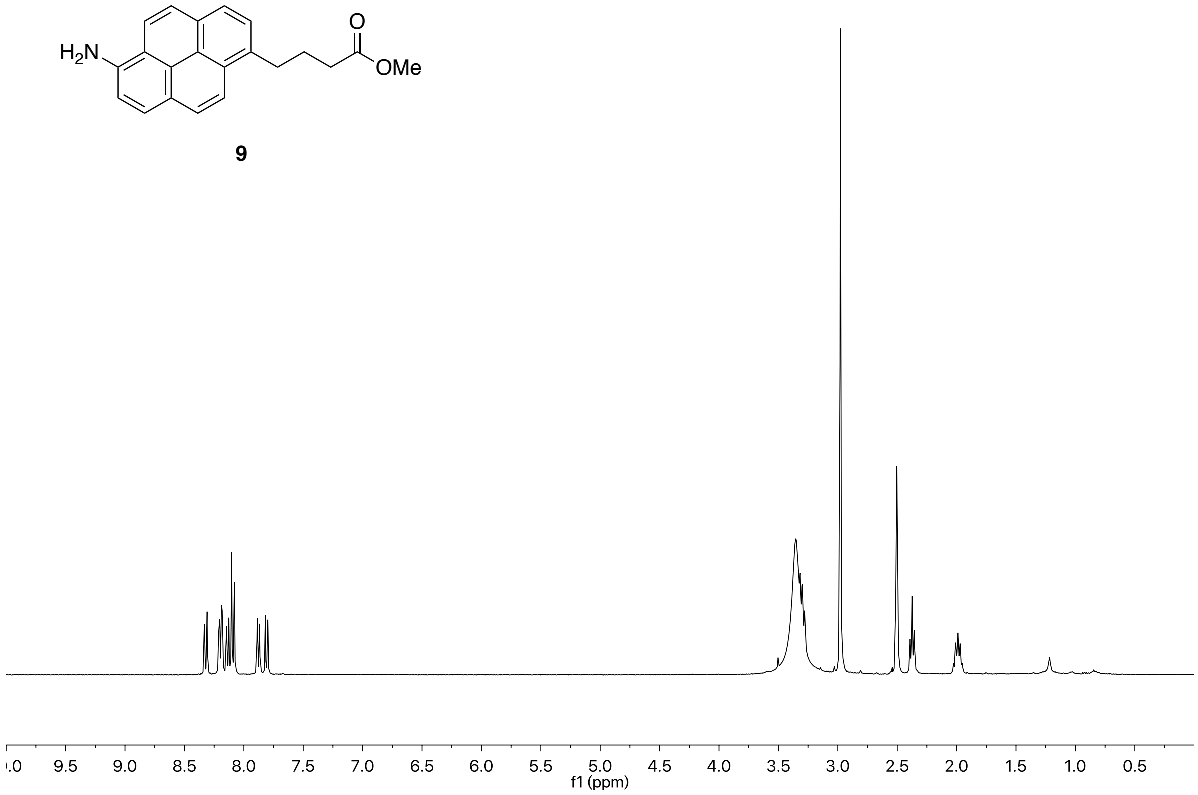

^{1}H NMR spectrum of **9** (400 MHz, DMSO-d_6).

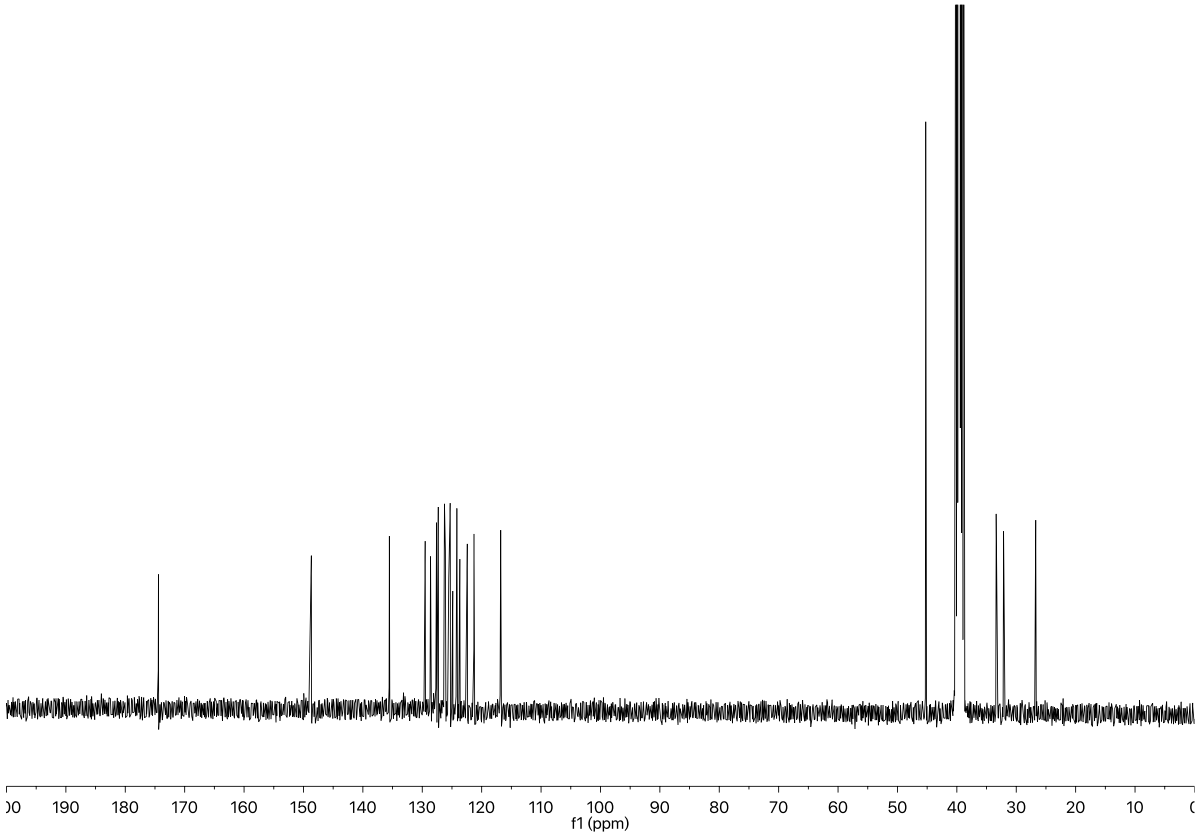

^{13}C NMR spectrum of **9** (100 MHz, DMSO-d_6).

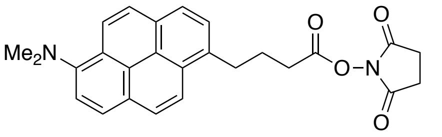

4

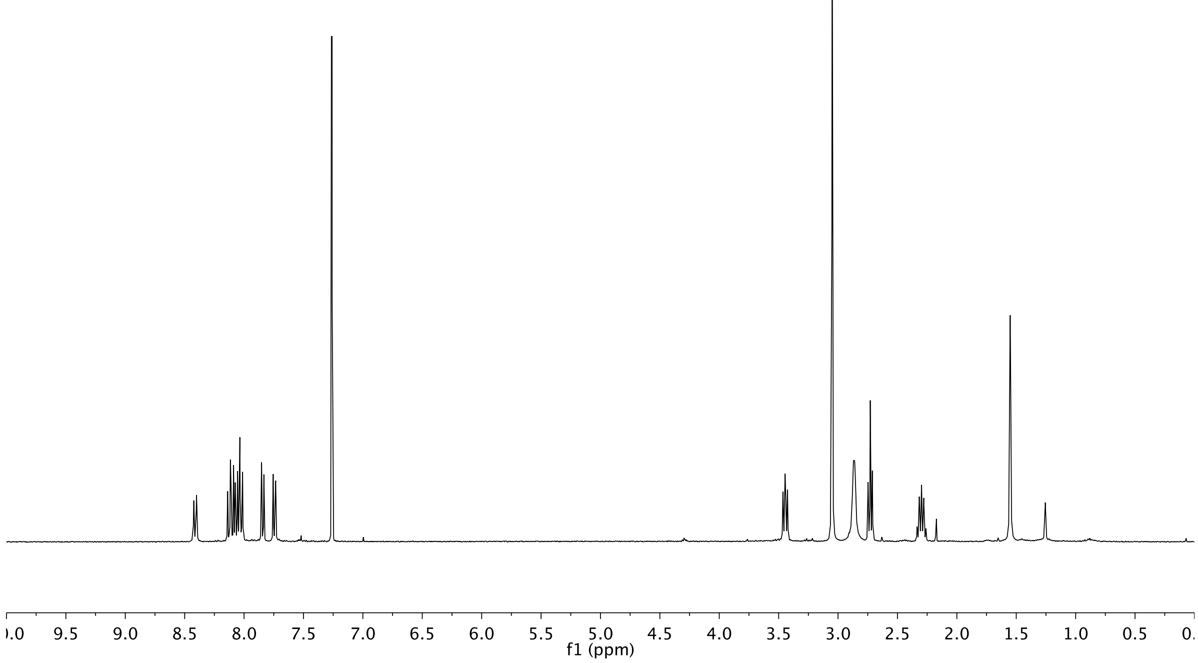

^{1}H NMR spectrum of **4** (400 MHz, $CDCl_3$).

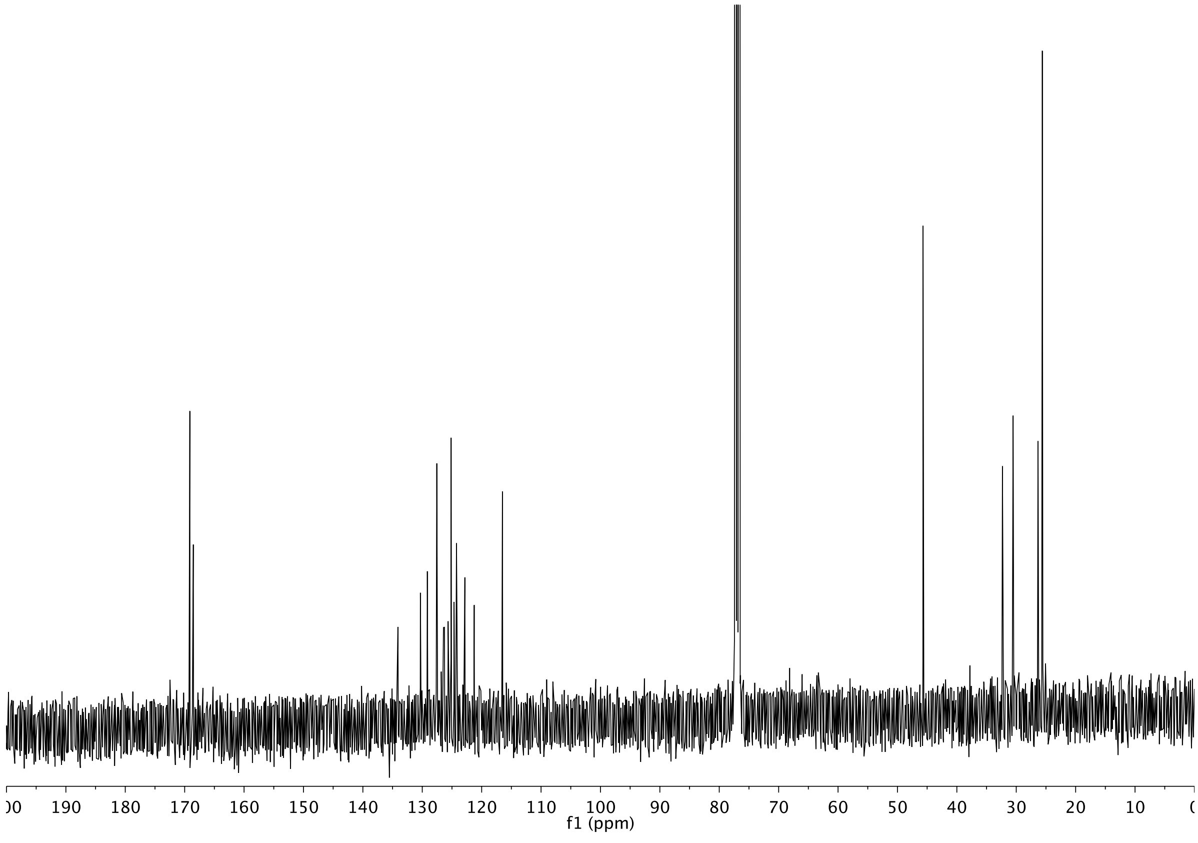

^{13}C NMR spectrum of **4** (100 MHz, $CDCl_3$).

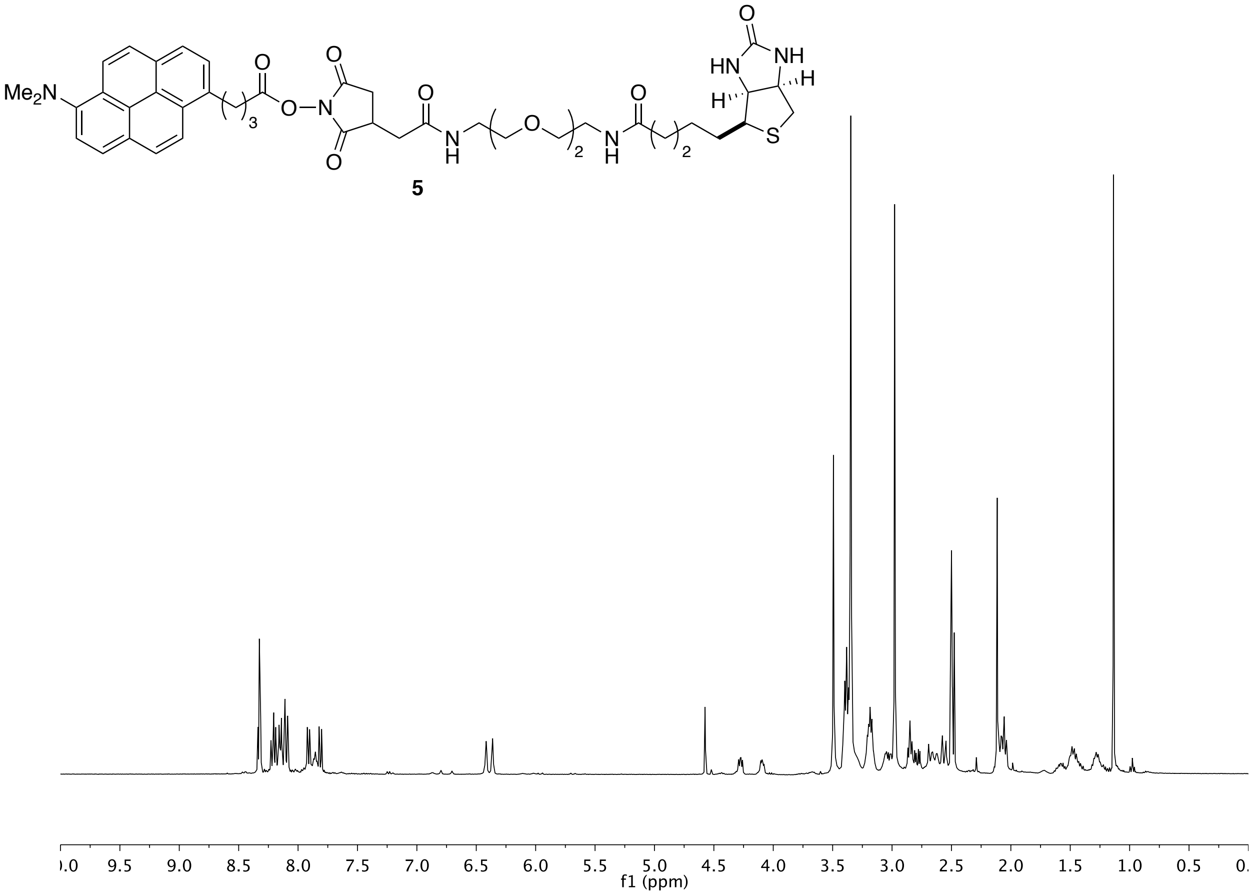

^{s1}H NMR spectrum of **5** (400 MHz, DMSO-d_6).

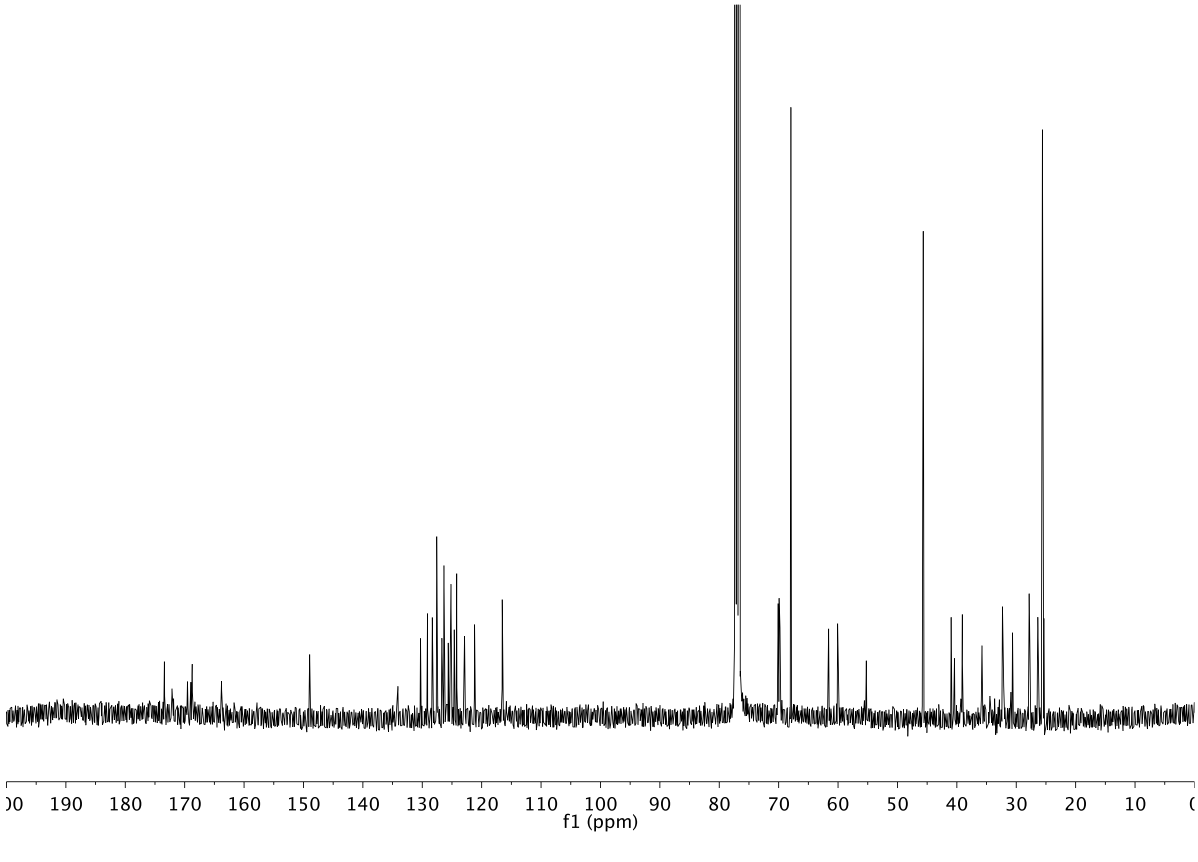

^{13}C NMR spectrum of **5** (100 MHz, $CDCl_3$).

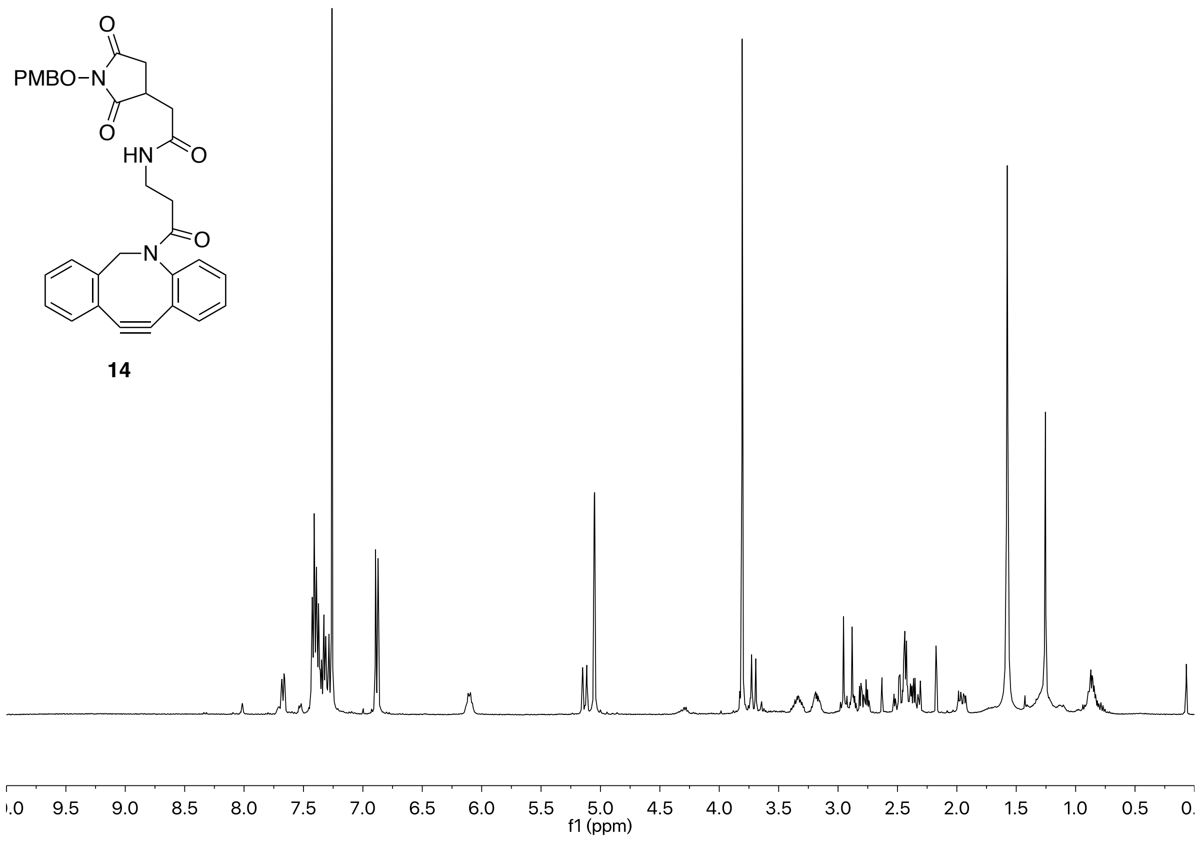

^{1}H NMR spectrum of **14** (400 MHz, $CDCl_3$).

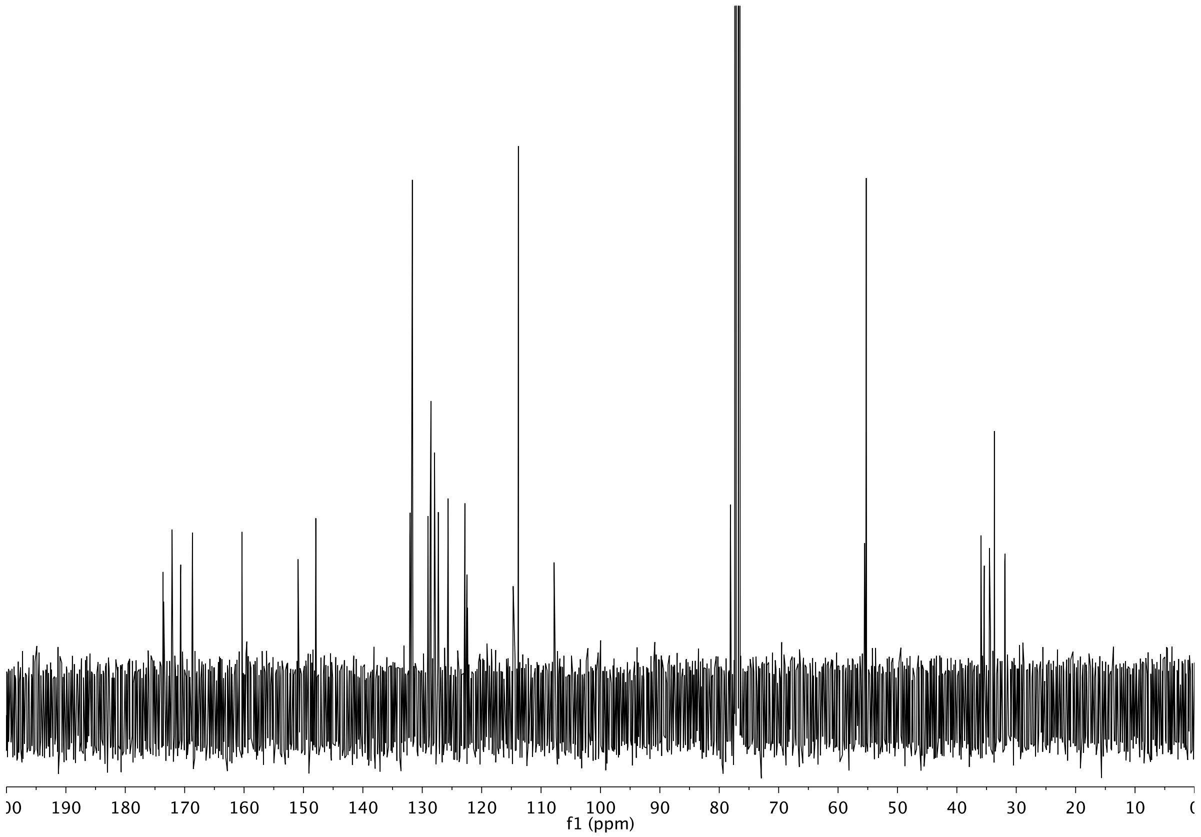

^{13}C NMR spectrum of **14** (100 MHz, $CDCl_3$).

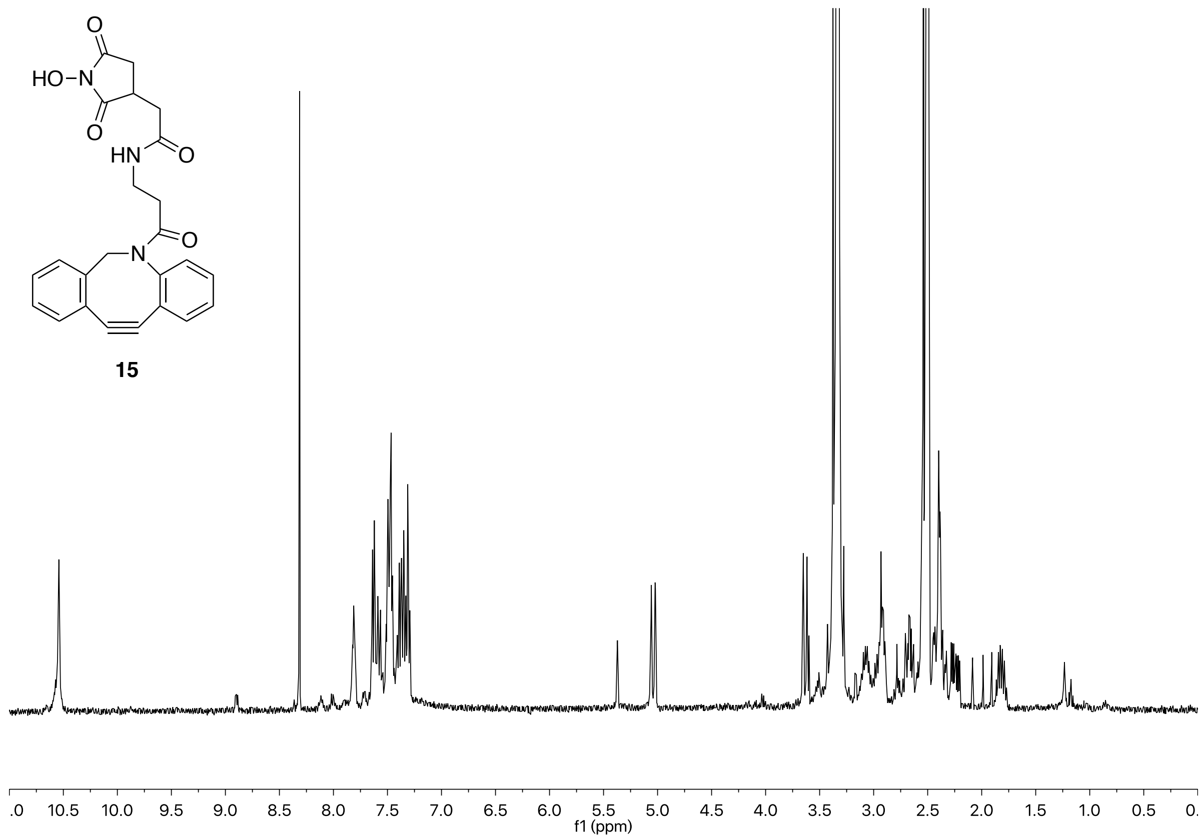

^{1}H NMR spectrum of **15** (400 MHz, DMSO-d_6).

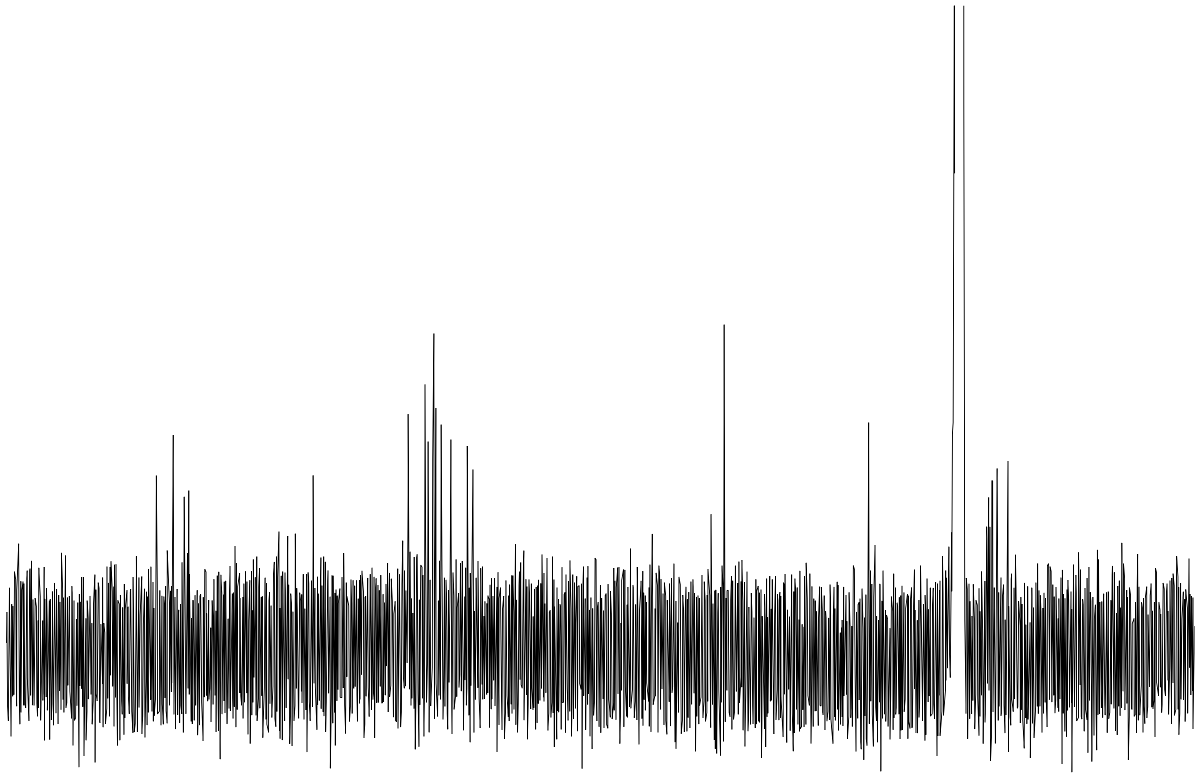

^{13}C NMR spectrum of **15** (100 MHz, DMSO-d_6).

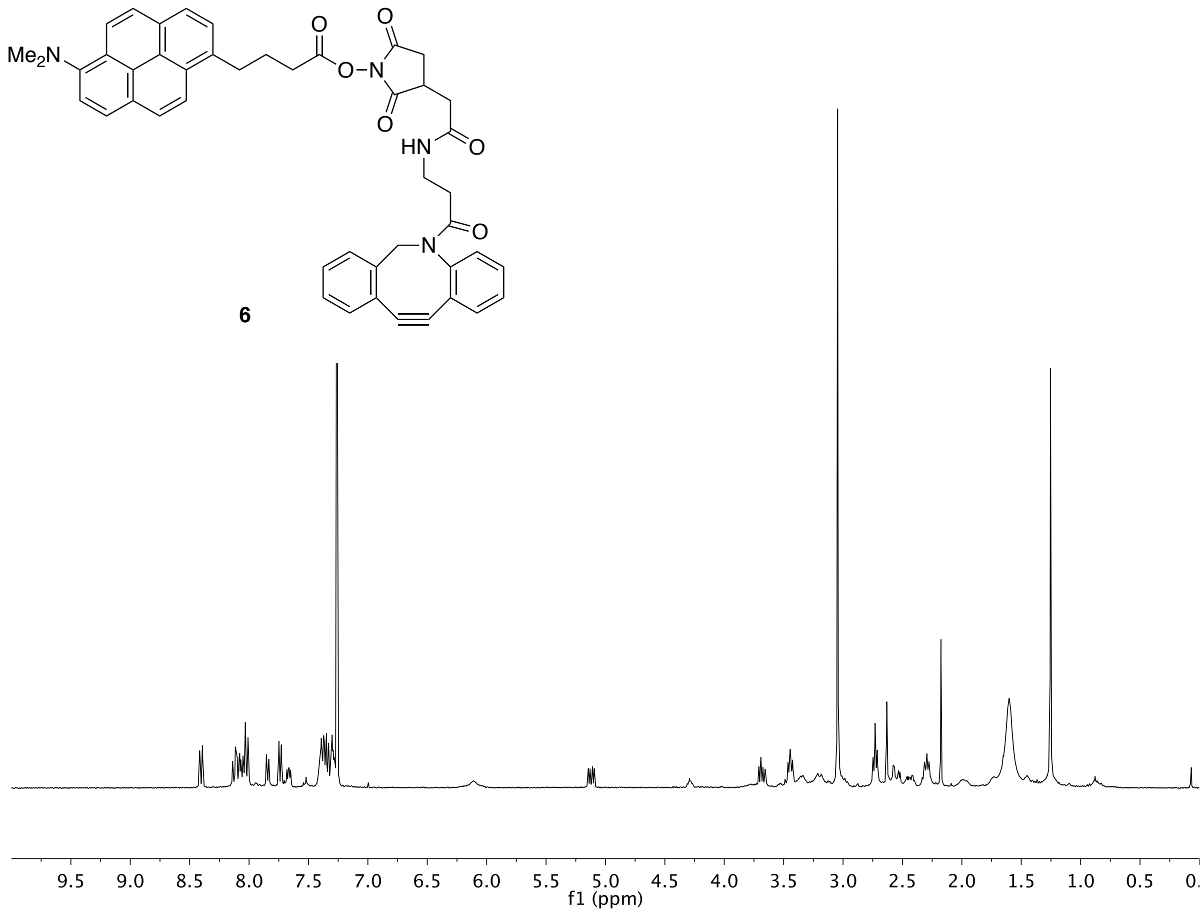

^{1}H NMR spectrum of **6** (400 MHz, $CDCl_3$).

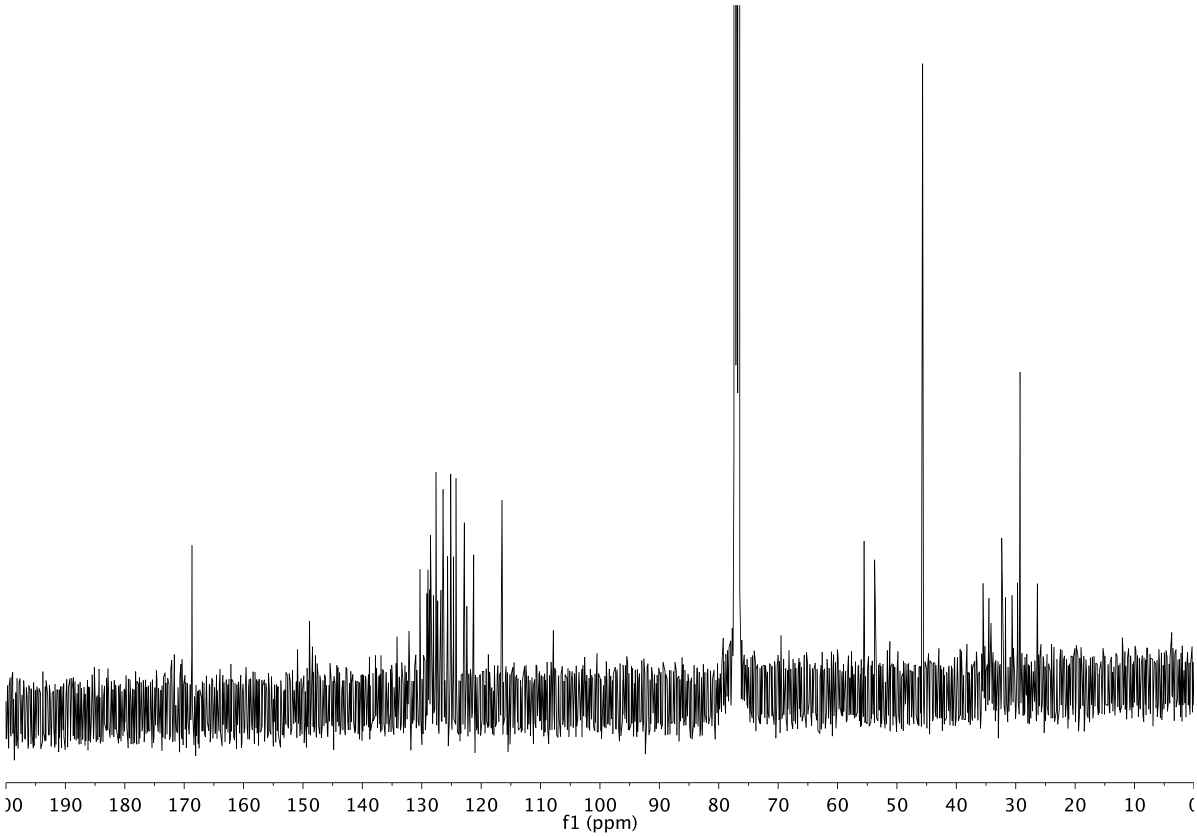

^{13}C NMR spectrum of **6** (100 MHz, $CDCl_3$).

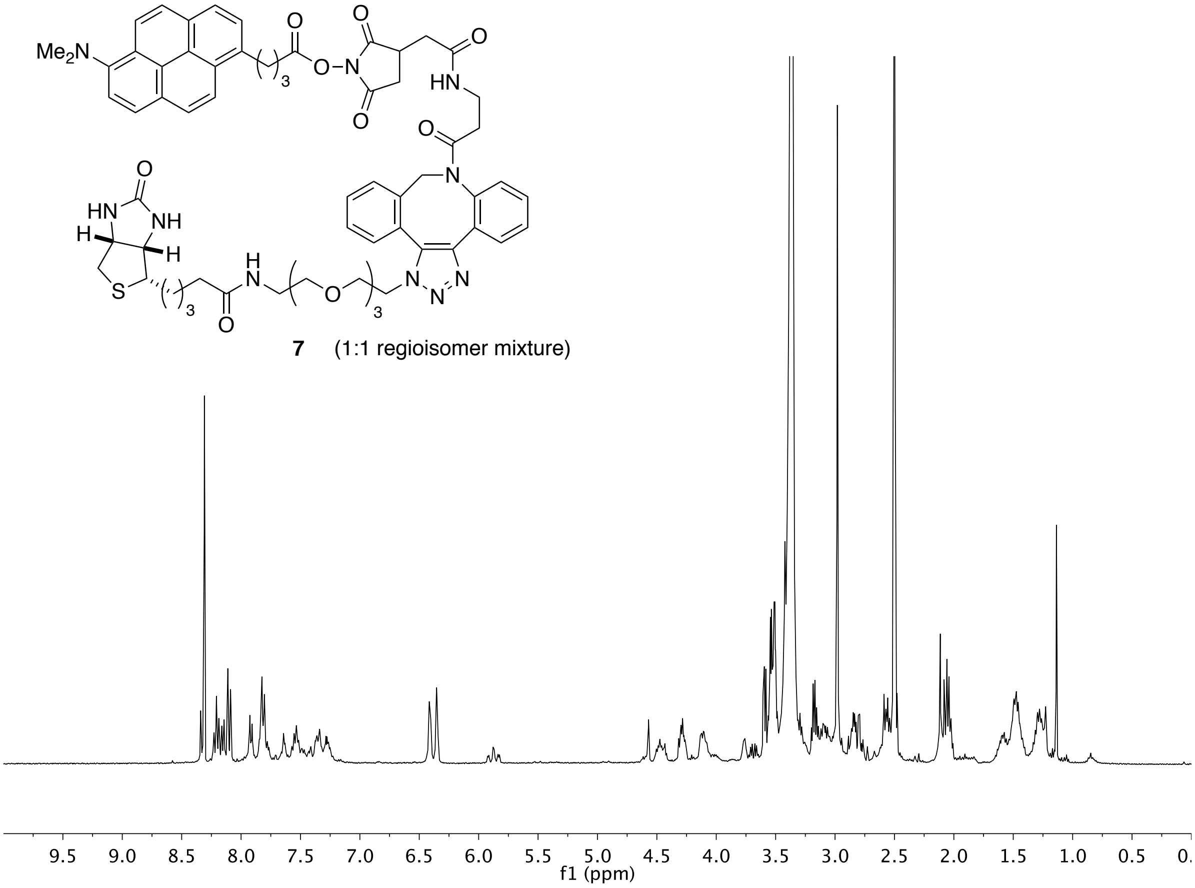

¹H NMR spectrum of **7** (400 MHz, DMSO-d_6).

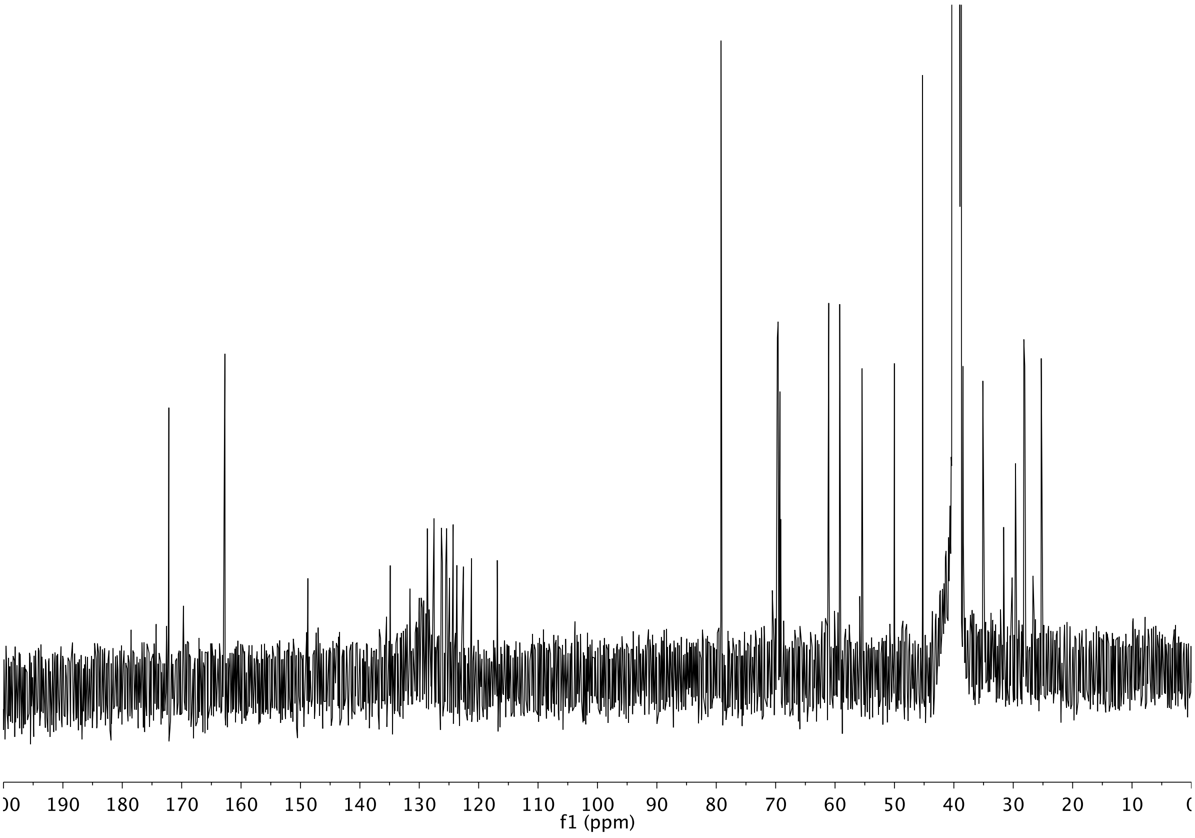

¹³C NMR spectrum of **7** (100 MHz, DMSO-d_6).

HPLC charts of synthetic dmpy- and apy-labeled peptides.

(a) **ANAWK(dmpy)STLVGHD** (Figure 2): t_R = 12.7 min.

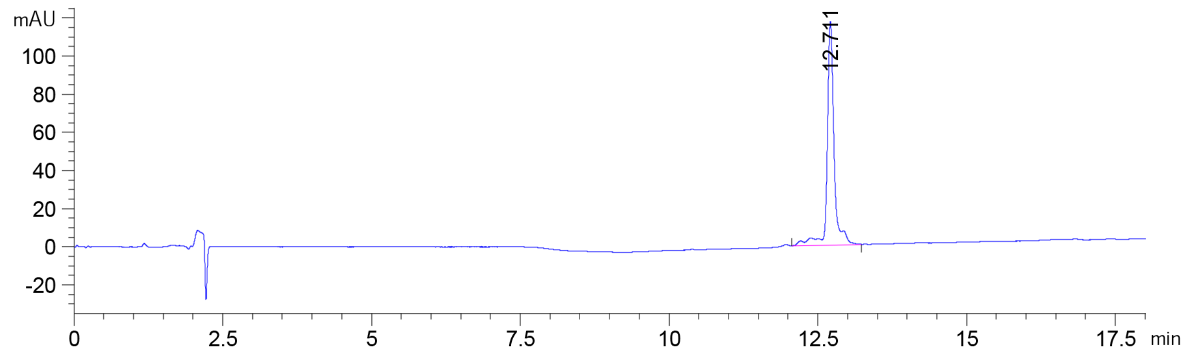

HPLC condition for (a): Column, Zorbax SB-C18 (0.5 mm I.D. × 150 mm); Eluate, 5% MeCN / 0.1% TFA for 3 min, 5–100% MeCN / 0.1% TFA for 15 min (linear gradient); Flow rate: 16 µL /min; Detection: UV 280 nm.

(b) **IK(dmpy)IIAPPER** (Figure 3a): t_R = 20.2 min.

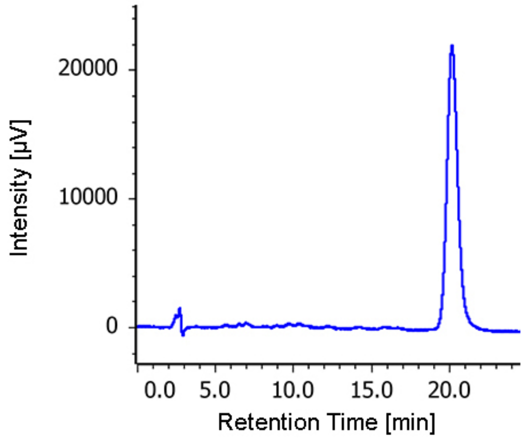

(c) **IK(apy)IIAPPER** (Figure 3b): t_R = 19.3 min.

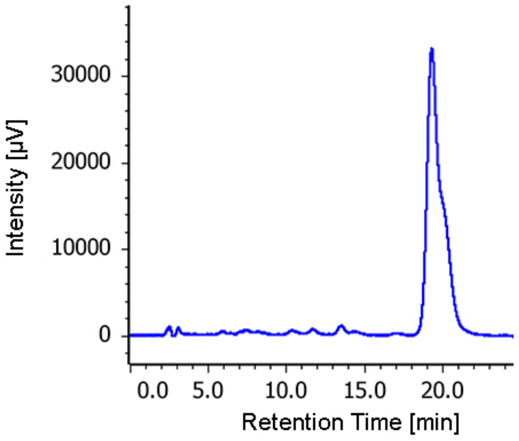

(d) **K(dmpy)ILTER** (Figure S2a): t_R = 14.3 min.

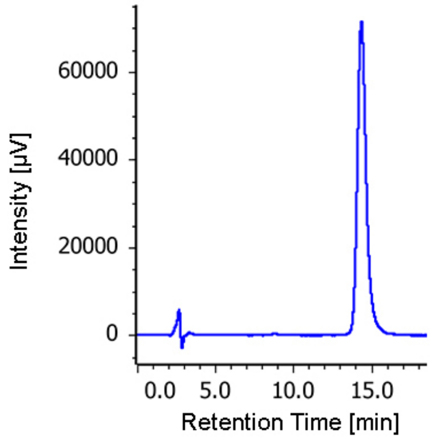

(e) **K(apy)ILTER** (Figure S2b): t_R = 16.7 min.

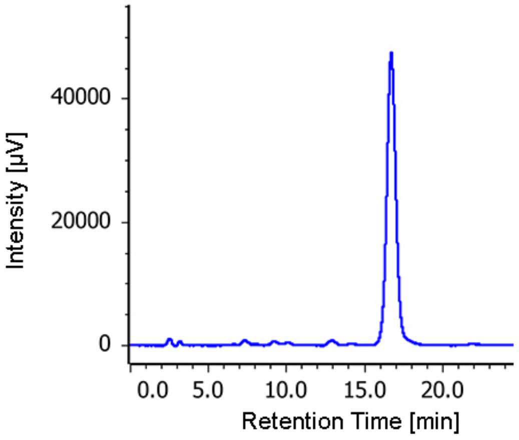

HPLC conditions for (b)–(e): Column, Develosil XG-C18LC (2.0 mm I.D. × 150 mm); Eluate, 30% MeCN / 0.1% TFA; Detection: UV 280 nm; Flow rate: 0.2 mL/min.
